# Micro Pattern‐Based 3D Cell Culture Platform: An Overview of Technologies and Applications

**DOI:** 10.1002/EXP.20240469

**Published:** 2026-02-24

**Authors:** Xinglong Zhu, Yi Li, Hulin Long, Yijia Wu, Ningwei Sun, Shun Li, Yiyao Liu, Huiqi Xie, Ji Bao

**Affiliations:** ^1^ Department of Pathology Institute of Clinical Pathology Key Laboratory of Transplant Engineering and Immunology West China Hospital Sichuan University Chengdu Sichuan China; ^2^ Department of Respiratory and Critical Care Medicine State Key Laboratory of Respiratory Health and Multimorbidity Institute of Respiratory Health Frontiers Science Center for Disease‐related Molecular Network West China Hospital Sichuan University Chengdu Sichuan China; ^3^ Department of Rehabilitation Medicine Hospital of Chengdu University of Traditional Chinese Medicine Chengdu Sichuan China; ^4^ Lammel Laboratory University of California Berkeley USA; ^5^ Molecular Cellular and Developmental Biology Department (MCD) Centre de Biologie Integrative (CBI) University of Toulouse CNRS UPS Toulouse France; ^6^ Department of Biophysics School of Life Science and Technology University of Electronic Science and Technology of China Chengdu Sichuan China; ^7^ Traditional Chinese Medicine Regulating Metabolic Diseases Key Laboratory of Sichuan Province Hospital of Chengdu University of Traditional Chinese Medicine Chengdu Sichuan China; ^8^ Department of Orthopedic Surgery and Orthopedic Research Institute Laboratory of Stem Cell and Tissue Engineering State Key Laboratory of Biotherapy West China Hospital Sichuan University Chengdu Sichuan China; ^9^ State Key Laboratory of Kidney Diseases Beijing China

**Keywords:** cell fates, cell spheroids, micropattern, organoids, 3D cell culture

## Abstract

Three‐dimensional (3D) multicellular models are considered ideal methods for bridging the gap between two‐dimensional (2D) cell culture and animal models, which are widely used in organogenesis, disease models, drug development, and regenerative medicine. Cell culture technologies determine the physical and biological properties of multicellular spheroids or organoids that affect the authenticity, stability, assessment, and throughput of the 3D multicellular system. Micro patterns, characterized as a coating of specific adhesion matrices on substrates, can control cell behaviors and fate by limiting the space available for cell spreading. micro patterns are used to culture non‐tumor or tumor spheroids and organoids with effective control of size and arrangement, which is suitable for large‐scale and standardized culture to generate 3D multicellular models. This comprehensive review summarizes the advantages and applications of 3D multicellular models and discusses the characteristics of general 3D cell culture technologies. We discuss the basic applications of micro pattern technologies and highlight the specific advantages and features of micropattern (as a 3D cell culture platform) in non‐tumor research (regenerative medicine, developmental biology, disease modelling, and monoclonal cell culture) and tumor research (tumor microenvironment (TME) and drug screening). Finally, the fabrication of micro patterns (bio inks, fabrication methods for micro patterns, morphology, and quality of micro patterns) is described.

## Graphical abstract

Micro patterns are characterized as a coating of specific adhesion matrices on substrates to support cell growth. An overview of micropattern technology to regulate single cell morphology and fates, further focusing on the micro pattern‐based 3D cell culture platform used in the non‐tumor and tumor researches.

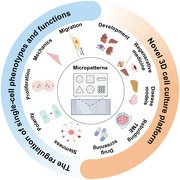

## 1 Introduction

For many years, traditional 2D cell culture has been used as an in vitro model for basic research, drug development, disease models, tumor research, regenerative medicine, and precision medicine due to its cost‐effectiveness and streamlined processes [1, 2]. The 2D model provides important basic in vivo pathophysiological information but cannot simulate the structure and physiological characteristics of in vivo tissue and lacks information on interactions and signal transduction between cells and the extracellular matrix (ECM) [3, 4]. 3D cell models, such as cell spheroids and organoids, have been widely used to bridge the gap between 2D and animal models [5, 6]. The advantages of 3D cell models over 2D cell models and their specific tissue properties have been broadly recognized [7]. Compared with 2D cell models, 3D cell models exhibit different morphological and physiological characteristics, which could more veritably reflect the conditions of tumors and accurately simulate the in vivo microenvironment [8, 9]. When cells are placed in a 3D environment, the transduction of cell signals and cell‐cell and cell‐ECM interactions are affected by the 3D spatial level rather than the 2D spatial level [10]. In addition, the 3D culture partly addresses the obstacles of the embryonic lethality of many genetic ECM knockouts and the limited resolution/imaging depth of intravital microscopy in in vivo models for the study of cell–ECM interactions [11, 12, 13]. Cell spheroids, as one of the simplest 3D models, can simulate many in vivo tissue characteristics, such as the interactions between multiple cells and the ECM, a hypoxic environment, a necrotic central core, and drug resistance, and have been widely used in disease models, regenerative medicine, tumor research and drug development [14, 15]. In recent years, the rapid development of organoids, especially patient‐derived organoids (PDOs), has provided new ideas for personalized treatment of patients [16, 17].

The application of 3D cell culture technology depends on whether it is precise, repeatable, affordable, and scalable, has high throughput and is amenable to microscopy or manipulation [9]. In general, traditional 3D cell culture methods, including the hanging drop method, micro well method, shaking technique, hydrogel (3D encapsulation) method, microfluidics, 3D bio printing, and electromagnetic, magnetic, and acoustic forces [6, 18, 19, 20, 21, 22, 23, 24, 25], can control the shape, size, surface features, internal textures, and density of cell spheroids individually or partially [26]. In particular, the shape, size, and arrangement of spheroids can affect the stability, efficiency, and standardization of experimental results and determine the reproducibility, observation, analysis and automated generation of cell spheroids [26, 27]. However, the above methods are unable to control the consistency of the cell spheroid shape, size, and ordered arrangement with cost‐effectiveness [28].

**FIGURE 1:**
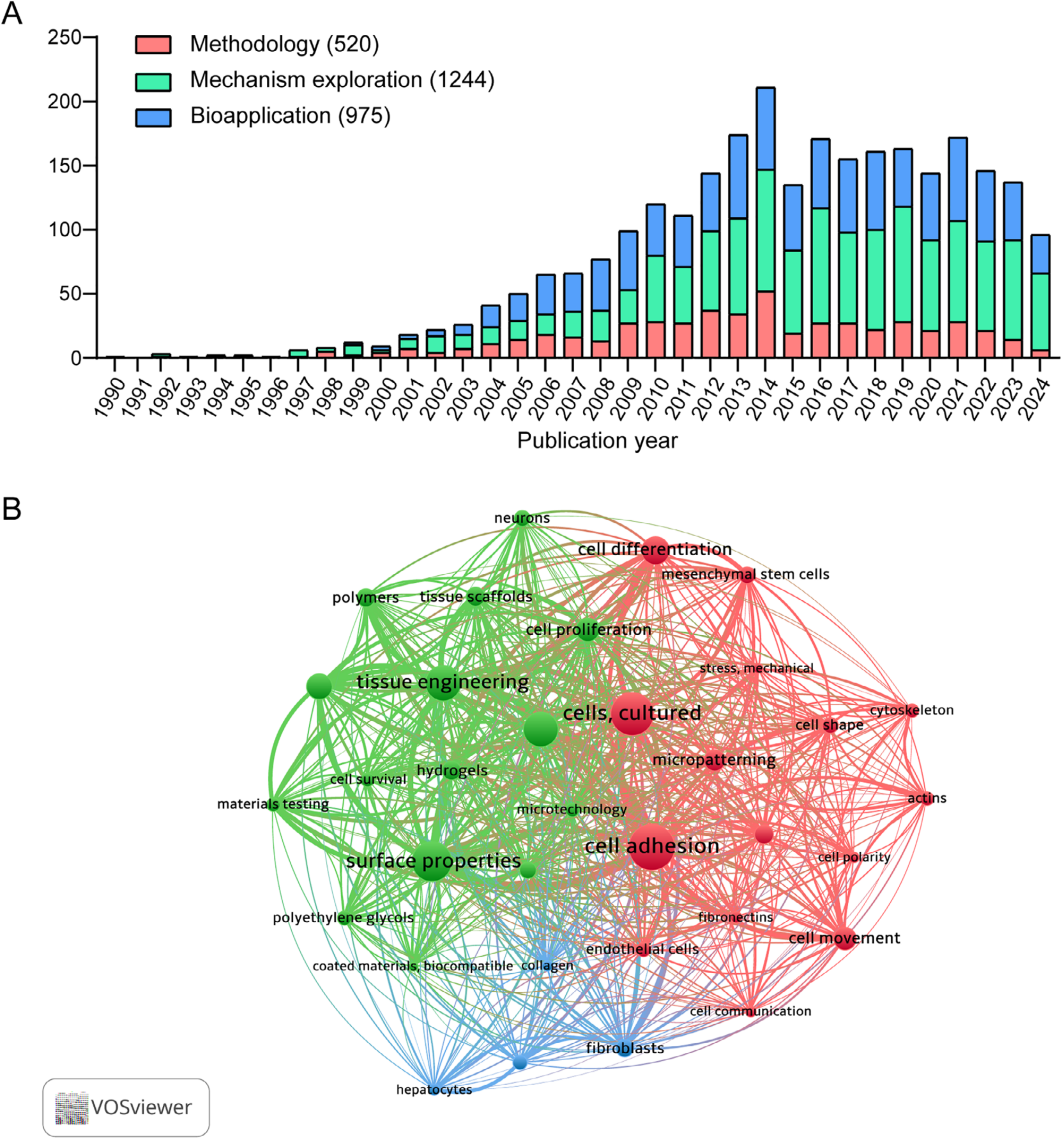
(A) Publication trends of micro pattern during 1990–2024; and (B) mapping and clustering of terms in publications related to micro pattern.

Micro patterns are a promising solution to these problems [29]. Specific adhesion matrices are used to generate micropatterns on cell culture substrates by micro‐nano fabrication technology. Cells are restricted to adhering to the micro patterned matrix, resulting in a limited space for cell growth. Furthermore, cells spontaneously assemble into spheroids with 3D multicellular structures through proliferation and cell‐cell adhesion [30, 31]. These arrangements ensure that the cultured cell spheroids have a controllable size and an orderly arrangement [27]. micro patterns have gradually become a powerful technology for controlling the cell position and morphology and are widely used to study cell polarity, proliferation, migration, and morphogenesis; the relationship between the cytoskeleton and cell functions; and cell stiffness and differentiation [32, 33, 34] (Figure 1). Micropatterns are promising for use in 3D cell culture, especially in large‐scale culture, standardized production, rebuilding of the TME and drug screening [29, 30, 31].

In this review, 3D cell models and general culture methods are briefly described and compared; the use of micropatterns as a technology for controlling cell morphology to regulate the cell phenotype is described and discussed; and the use of micro patterns for culturing cell spheroids, especially for mimicking the TME and for drug screening, is summarized. Finally, the fabrication of micro patterns is described, including bio inks, fabrication methods, and micro pattern shapes.

## 2 3D Cell Models and General 3D Cell Culture Technologies

**FIGURE 2:**
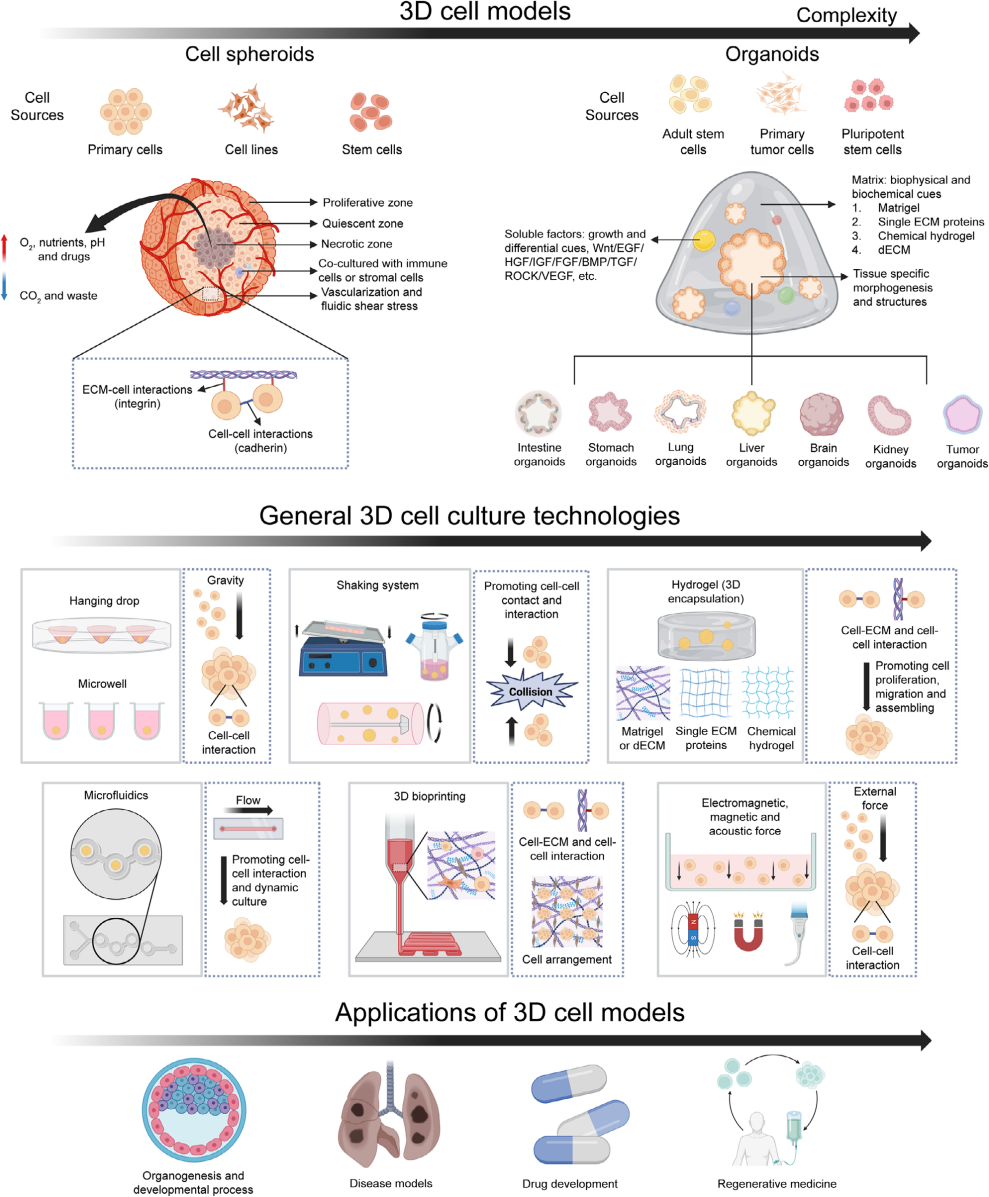
General cell culture technologies for 3D cell models (cell spheroids and organoids) to biomedical applications.

3D cell culture strategies have unique advantages due to the provision of multicellular spatial structural features and physical environments similar to those in vivo over 2D cell culture (Table 1) (Figure 2). Generally, 3D cell models could be divided into cell spheroids and organoids.

**TABLE 1:** Characteristics of the three mainstream in vitro cell models.

Types of in vitro cell model	2D	3D		References
		Cell spheroids	Organoids	
Basic characteristics	—	—	—	—
Cost	+	++	+++	[56]
Time demand	+	++	+++	[7]
Ease of operation	+++	++	+	[40]
Large‐scale culture	+++	++	++	[42]
Long‐term culture	+	+++	+++	[7]
High throughput	+++	++	++	[18]
Cell type	+++	++	++	[46]
Representation of in vivo tissue	—	—	—	—
3D structure and microenvironment	—	++	+++	[57]
Cell‐cell and ECM interaction	+	++	+++	[10]
Cell polarity	+	++	+++	[17]
Biological factor diffusion	+	+++	+++	[17]
Dynamic microenvironment	+	+++	+++	[58]
Physiological functions	+	++	+++	[10]
Heterogeneity	+	++	+++	[49]
Mutability	+++	++	+	[9]
In vivo similarity	+	++	+++	[47]
Biomedical applications	—	—	—	—
Developmental biology	+	++	+++	[8]
Disease model	+	++	+++	[1]
Drug evaluation and screening	+	++	+++	[59]
Regenerative medicine and tissue engineering	+	++	+++	[60]

Cell spheroids exhibit morphology, polarity, behavior, movement, growth, differentiation, gene expression, protein expression, and sensitivity to radiotherapy and chemotherapy that are more similar to those in physiological conditions [35, 36]. Moreover, having the characteristic of a multicellular structure of, cell spheroids can reproduce the dense 3D structure of solid tissue in vitro [37] and can exhibit multi‐layered architectures but with different characteristics at an appropriate size [38], including an outer proliferative layer with active cell proliferation, an intermediate quiescent layer without proliferative activity but with exhibits high cell viability, and an inner necrotic core characterized by necrotic cell death [4, 38]. Because of the limited diffusion of oxygen and nutrients, from outside layers of the cell spheroids with multilevel cell geometrical structures, the oxygen, pH, and drug concentration decrease inwards [39, 40]. This biochemical gradient is a double‐edged sword for cell therapy when the spheroid diameter exceeds 100 µm: on the one hand, it is unfavorable for the viability of primary hepatocyte or mesenchymal stem cells (MSCs) spheroids [20, 41, 42]; on the other hand, the hypoxic environment may enhance the secretion of proangiogenic factors or stem cell differentiation [43, 44]. Advantageously, the multilevel geometrical structure of cell spheroids and limited supplies of oxygen and nutrients more accurately simulate the characteristics of in vivo tumor tissue [38]. Consequently, the optimal size of cell spheroid should be determined by the intended application.

Organoids derived from normal tissues, including the small intestine, lung, liver, and brain, have been successfully established and are used in organogenesis, basic research, regenerative medicine, and disease models [1, 45]. It is noteworthy that PDOs are showing their great potential value in personalized medicine [16, 46]. In terms of drug screening, the feasibility and stability of PDOs can be described in three aspects. Firstly, the heterogeneity of the primary tumor can be maintained during the long‐term culture of PDOs. Secondly, the results of drug screening in PDOs are consistent with those observed in clinical diagnosis and medication. Thirdly, the heterogeneity of PDOs as a “cell line” can be maintained in the subculture process, which guarantees the stability of drug screening results [47, 48, 49]. Nevertheless, the extensive and pervasive use of PDOs in drug screening still faces several challenges. Firstly, the cost of culturing PDOs is high and the process is complex. Secondly, it is difficult to culture PDOs from some tumor types; for instance, the success rate of culturing PDOs from breast cancer is very low. Thirdly, the differentiation and variation of PDOs may occur during long‐term culture. Fourthly, there are limitations to obtaining tissue from living tumors by resection or biopsy. Fifthly, natural ECM, such as Matrigel, is required to encapsulate tumor cells in the culture of PDOs. However, different batches of Matrigel derived from the Engelbreth‐Holm‐Swarm mouse sarcoma are unstable, which could limit the standardized establishment of PDOs [50, 51, 52, 53].

At present, a variety of 3D cell culture technologies have been developed (Table 2). For the induction of cell‐cell adhesion and cell proliferation, followed by self‐assembly to generate spheroids, several traditional culture methods, such as the hanging drop method, micro well method, and rocked suspension culture, have been used in the culture of various cell spheroids [15, 54]. Along with multidisciplinary development and cross‐integration, many novel 3D cell culture technologies, such as matrix encapsulation culture, microfluidics, magnetic levitation, and 3D printing, have focused on simulating tissue‐specific microenvironments [6, 9]. Matrigel is utilized to encapsulate tumor cells for spheroid generation, or spheroids can provide a natural 3D ECM microenvironment [17, 55]. The combination of microfluidics with hanging drops and microwells was used to simulate blood flow to obtain a dynamic culture [21, 38, 55]. However, 3D cell culture technologies determine the shape, size, density, and surface structure of cell spheroids, which can affect the authenticity, stability, assessment, and throughput of drug screening [26]. Consequently, it is important to standardize and achieve the homogeneity of cultured cell spheroids; this could be solved by using micro pattern array chips, which represent a novel 3D cell culture method.

**TABLE 2:** Advantages, disadvantages, and applications of 3D cell culture technologies.

Technologies	Advantages	Disadvantages	Applications	References
**Hanging drop**	Simple operation Low cost High success rate Coculture of multiple cell types	Hard to replace culture medias Difficult to achieve long‐term culture Inhomogenous cell spheroids Difficult to observation and analysis	Culturing cell spheroids or organoids for regenerative medicine Studying cell‐cell interactions Drug screening	[6, 55, 61, 62]
**Microwell method**	Simple operation Low cost Coculture of multiple cell types Uniform shape and size of the spheroids Forming microwell array Large‐scale culture	Labor intensive Difficult to achieve long‐term culture Limited the paracrine signaling between the spheroids	Culturing cell spheroids or organoids for regenerative medicine Studying cell‐cell interactions Drug screening	[15, 63, 64, 65, 66]
**Shaking system**	Simple operation Large‐scale culture Long‐term culture Dynamic culture	Needing specific equipment Inhomogenous cell spheroids Generating fluid shear force to affect cell morphology and viability	Culturing primary hepatocytes, MSCs and tumor spheroids	[20, 54, 67, 68]
**Hydrogel (3D encapsulation)**	3D scaffolds to support cell culture Interaction between cells and ECM Simulating in vivo microenvironment	Labor intensive High cost Inhomogenous cell spheroids or organoids Difficult to observation and analysis	Culturing cell spheroids and organoids Studying cell‐ECM interactions Rebuilding ecological niche and TME Carrying cells for tissue engineering	[16, 19, 69, 70, 71]
**Microfluidics**	Large‐scale culture Long‐term culture Dynamic culture High‐throughput	High cost Needing specific equipment	Studying hemohydrodynamics, interstitial fluid hydrodynamics and TME High throughput drug screening	[21,]^38^, ^72^,^73^]
**3D bioprinting**	Controlling multi‐type cell arrangement Fabricating specific structure	High cost Needing specific equipment Phototoxicity	Construction of bioengineering tissues and organs for regenerative medicine Rebuilding the TME and tissue‐specific microenvironment	[9, 22, 74, 75]
**Electromagnetic, magnetic and acoustic force**	Large‐scale culture Accelerating culture speed	High cost Needing specific equipment Electricity, magnetic nanoparticles and acoustics affecting cell viability and functions Magnetic nanoparticles influencing colorimetric detection	Culturing stem cell spheroids and tumor spheroids	[23, 24, 25, 54]

## 3 Micro Pattern Technology

**FIGURE 3:**
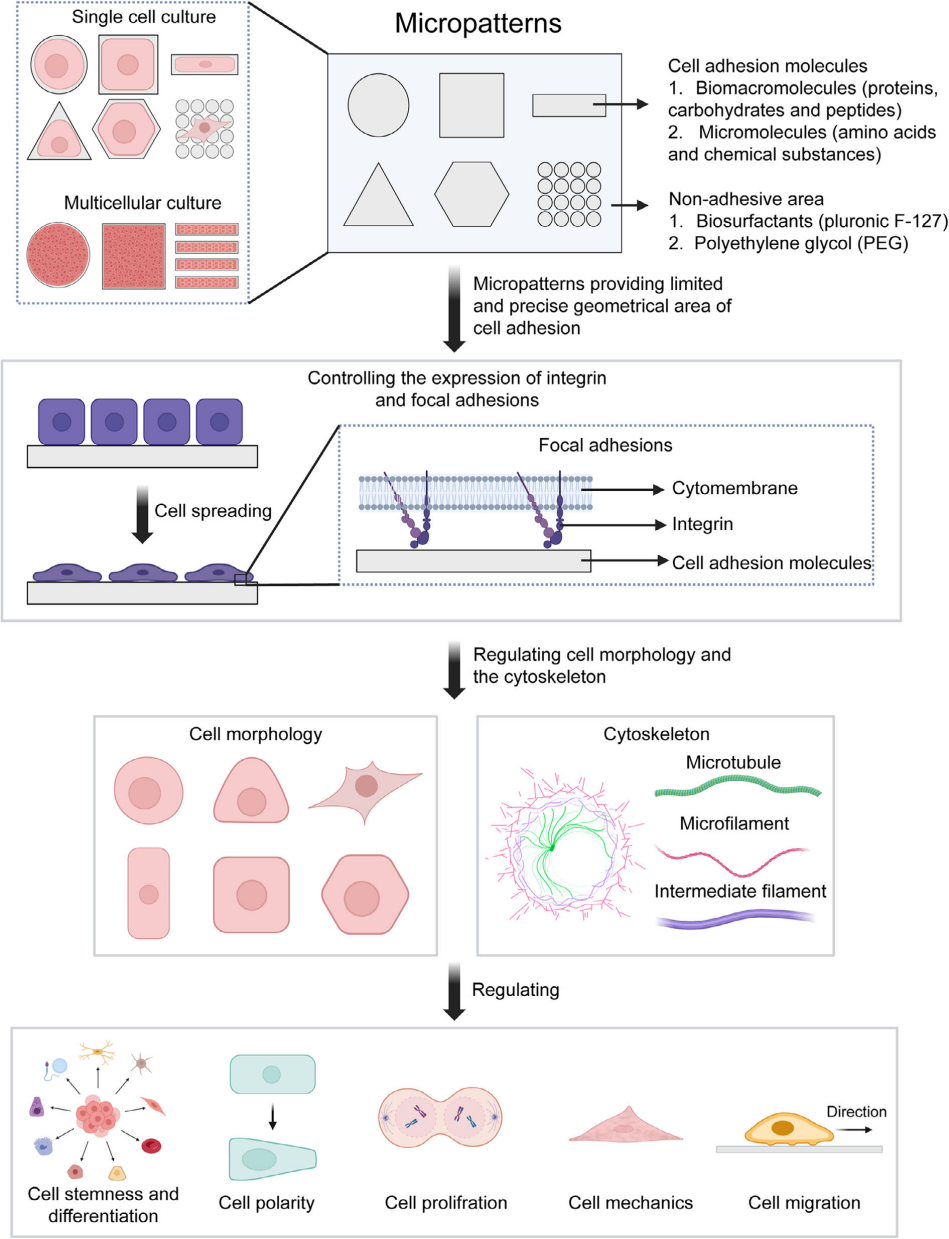
Micropattern technology as a potent single‐cell platform for manipulating cellular phenotypes and functions.

**FIGURE 4:**
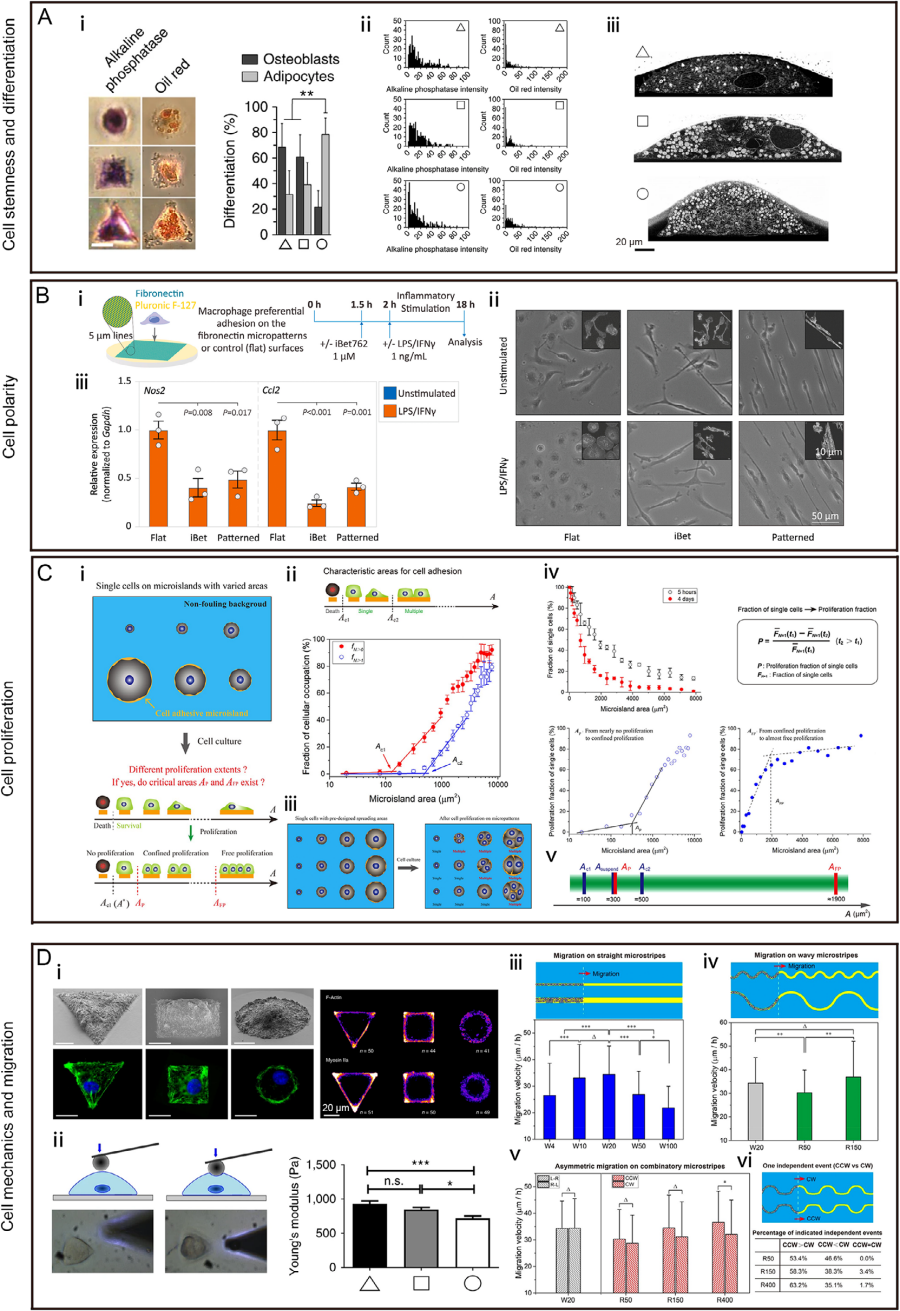
Micro pattern regulated cellular phenotypes and functions, including stemness, differentiation, polarity, proliferation, mechanics, and migration. (A) Micro pattern regulated MSCs stemness and differentiation: (i) Representative images showed MSCs differentiated into either fat (OilRedO) or bone cells (alkaline phosphatase activity) on triangular, square, and circular micro patterns, and the adipogenic and osteogenic differentiation of micro patterned hMSCs was quantified; (ii) frequency histograms of OilRedO and alkaline phosphatase activity; and (iii) representative images of whole‐cell cross‐sections of MSCs. Reproduced with permission [77]. Copyright 2018, Springer Nature; (B) elongation of cells by micro pattern drives macrophage polarization: (i) Schematic of generating micro pattern (arrays of fibronectin lines) and seeding cells. Phase contrast images (ii) and relative M1 marker (Nos2 and Ccl2) expression (iii) of macrophages cultured on the flats, iBet flats and micro patterned substrates. Reproduced with permission [78]. Copyright 2021, Elsevier. (C) Proliferation behavior of single MSCs on micro patterns with different size: (i) Schematic presentation of the idea to explore the effects of spreading areas on proliferation of single cells and corresponding critical areas; (ii) fraction of cellular occupation per micro pattern and corresponding characteristic areas for MSC adhesion; (iii) schematic presentation of proliferation of single cells with varied confined spreading; (iv) statistical results of the proliferation fraction of single cells per micro pattern; (v) schematic presentation of the relative size relationship of different kinds of characteristic areas for MSCs proliferation. Reproduced with permission [79]. Copyright 2019, American Chemical Society; (D) micro pattern regulates cell mechanics and migration: (i) representative SEM micrographs, F‐actin staining and immunofluorescence staining (myosin IIa); (ii) live cell stiffness measurement of MSCs cultured on triangular, square and circular micro patterns. Reproduced with permission [77]. Copyright 2018, Springer Nature; (iii) schematic presentation of cell migration; (iv) statistical results of migration velocity on straight micro stripes (width of 4, 10, 20, 50, and 100 µm) or wavy micro stripes (width of 20 µm yet varied arc radiuses of 50 and 150 µm); (v) statistical results of clockwise and counterclockwise migration velocities of cells on combinatory micro stripes of the same width of 20 µm yet with arc radius of 50, 150, and 400 µm; and (vi) schematic presentation of one independent event for the comparison of clockwise and counterclockwise migrations on two adjacent combinatory micro stripes with the same arc radius and corresponding statistical results of the percentage of independent events. Reproduced with permission [80]. Copyright 2020, American Chemical Society.

Micro patterns are generated by micro fabrication techniques used to coat a matrix onto substrates; these techniques fabricate a precise geometrical area with adhesive ECM that restricts the range of cell adhesion and spreading [33, 34, 76]. Using micropattern technology as a potent single‐cell control platform, cells are positioned in a confined area with the adaption of integrin/FAs signaling, which further controls cell morphology and the cytoskeleton through mechanotransduction, and subsequently regulates cellular phenotypes and functions, including stemness, differentiation, polarity, proliferation, mechanics, and migration (Figures 3 and 4).

### 3.1 Control of Cell Morphology and the Cytoskeleton by Cell Adhesion

Cell adhesion is tightly associated with embryogenesis and tissue engineering [33]. The adhesion between cells and the ECM is dependent on several different trans membrane adhesion receptors, including integrins, cadherins, selectins, and immunoglobulin‐like cell adhesion molecules [81, 82]. In most cell types, integrins, as a critical component of the focal adhesion complex, play a determinant role in cytoskeleton attachment to the ECM and are associated with mechanical force sensing and transduction [83, 84]. Cells apply traction stress on the ECM, which can regulate the stabilization of FAs and the cytoskeleton, thus guiding diverse biological activities [33]. The expression of integrins is affected by the micropattern geometry. Lee et al. reported that when MSCs were cultured on micropatterns, the expression of integrin's α3, α5, αv, α6, β1, and β3 was greater in star‐shaped cells than circular shaped cells [85]. In addition, FAs could be affected by the concentration of integrin ligands which are provided by the ECM. Micro patterns fabricated with a lower concentration of fibronectin were shown to decrease the quantity of FAs and subsequently regulate cytoskeletal tension [33, 86, 87]. Cell morphogenesis is an important biological process that involves cell adhesion, rearrangement, spreading, competition, proliferation, and migration, which is controlled by the cell shape and further regulates cell functions [34, 88]. Micropatterns that are arranged in specific geometrical shapes, including circles, squares, triangles, and polygons, regulate the cell shape and morphogenesis by controlling cell adhesion, cell spreading, the cytoskeleton, cell roughness and subcellular curvature [33]. This mechanical control derived from cellular morphological confinement is thought to be regulated by the YES‐associated protein (YAP) [89]. Focal adhesion formation and cytoskeletal remodeling can induce nuclear flattening and nuclear pore stretching, thereby increasing YAP nuclear import [90, 91].

### 3.2 Cell Stemness and Differentiation

Micro patterns can be used to modify the stemness and differentiation properties of stem cells. Cell geometry, modulated by micro patterns, could regulate the nanostructure of the plasma membrane and trigger signaling events involving the serine/threonine kinase Akt/protein kinase B (PKB) that affect stem cell fate. Triangular MSCs showed higher osteogenic differentiation, while circular MSCs exhibited higher adipogenic differentiation [77]. Wang et al. reported that the stemness of MSCs could be influenced by micro patterns with different shapes, sizes, and aspect ratios (ARs). The expression of stem cell markers (CD44, CD73, CD105, and CD106) decreased as the spreading area and AR increased through improving the activation of nuclear activities and elongation of the cytoskeleton. However, the stemness of MSCs was hardly influenced by the shape of the micro pattern [92]. Cell differentiation is also affected by the confined of the cell shape with micro pattern technology. Kilian et al. demonstrated that when MSCs were cultured on different micropatterns with areas of 1000, 2500, and 5000 µm^2^, small micro patterns were favorable for adipogenic differentiation, while large micro patterns were beneficial for osteogenic differentiation. By increasing the ARs of the micro patterns, osteogenesis was promoted. Geometric restrictions regulated the cytoskeleton and increased actomyosin contractility, which promoted osteogenesis by activating c‐Jun N‐terminal kinase (JNK) and extracellular signal‐regulated kinase (ERK1/2), and increasing wingless (Wnt)‐type signaling [93]. Moreover, Peng et al. compared the effects of square and rectangular micro patterns with different ARs on MSC differentiation. Cells in square micro patterns showed predominantly adipogenic differentiation, whereas cells in rectangular micro patterns with an AR greater than two exhibited osteogenic differentiation [94]. Micro patterning has also been used to investigate the mechanistic induction of stem cell differentiation by mechanical stimulation. Recently, substrates with micro patterned lines have been used to control membrane and nuclear curvature to investigate the direct role of curvature on MSC differentiation, revealing the direct role of curvature on YAP activity driven by both active and passive nuclear import [95].

### 3.3 Cell Polarity

Cell polarity affects cell growth, development, communication, and functions, as well as pathological processes [96, 97]. Characteristics of cell polarity mainly involves the asymmetry in the morphology, cytoskeleton, organelles, focal adhesion, migration, and arrangement [34], which can be manipulated by micropatterns. Lee et al. fabricated circular micropatterns with diameters of 1, 3, and 5 µm to modulate the intracellular polarity of mouse embryonic fibroblasts. Analysis of changes in the relative positions of the nucleus and microtubule‐organizing center showed that cell polarity was regulated by the cell‐ECM adhesion and the interaction between the nucleus and cytoskeleton. The degree of cell polarity was decreased by disrupting nuclear lamin A/C [98]. Furthermore, Costa et al. utilized micro pattern technology to understand collective epithelial cell polarity. Collective cell polarity was found to be associated with asymmetric micro patterns, and the degree of polarity was regulated by the degree of asymmetry and calcium‐dependent cell adhesion through the control of the microtubule network [99].

Physical cues present essential roles in the polarization of macrophages [100], while soluble factors are generally considered the major regulators of macrophage polarity in vitro. Micro pattern technology has been used to understand mechanical cues affecting macrophage polarity [101]. McWhorter et al. demonstrated that, without exogenous cytokines, 20‐µm line micro patterns resulted elongation of macrophages could induce their polarization (M2 phenotype) and reduced the secretion of inflammatory cytokines. This cell geometry‐induced macrophage polarity can be suppressed by the inhibition of actin and actomyosin contractility [100]. Veerasubramanian et al. further studied the mechanism of biomechanics induced macrophage polarization. Although inflammatory stimuli (LPS/IFNγ) improved the marker expression of traction forces, Src activity, and H3 acetylation (H3Ac) and reduced the cell elongation and motility in macrophages. However, through micro patterns (5 µm lines) inducing the elongation of macrophages, these effects were curtailed by disruption of H3Ac‐signaling [78].

### 3.4 Cell Proliferation and Division

As the micropattern determines cell morphology, cell proliferation, and apoptosis can be further impacted [33]. Generally, the proliferative ability of cells on large‐size micro patterns was greater than that on small‐size micro patterns [33]. Cells cultured on large‐area micro patterns went through the G1 and S phases by upregulating the expression of cyclin D1 and down regulating that of the cyclin‐dependent kinase inhibitor p27^kip1^[33, 102, 103]. Yao et al. systematically reported that the proliferation of stem cells, tumor cells and normal cells cultured on micropatterns was dependent on cell adhesion and the spreading area [79]. Not only the cell spreading area, but also the cell shape affects cell proliferation. Tong et al. demonstrated that circles were more beneficial for osteoblast proliferation than rectangles, triangles, and squares, which were dependent on the nuclear shape index but not the cell shape index. The concentration of Ca^2+^ increased with the nuclear shape index owing to the up regulated expression of inositol 1,4,5‐triphosphate receptor 1 and sarco/endoplasmic reticulum Ca^2+^‐ATPase 2, which were related to the intracellular calcium transient. Finally, cell proliferation was determined by the intracellular calcium transient [104]. In addition, micropatterned microstructures can be used to study the regulation of biophysical and biochemical niche signals on cell behavior and fate, and Huang et al. use microarrays of protein‐based 3D single cell micro‐niche show that the cell division direction is controlled by the biophysical niche signals in a cell shape‐independent manner [105].

### 3.5 Cell Mechanics

Micropatterns can manage cell stiffness by modulating the cytoskeletal stiffness, which increases as cells elongate and spread [92]. The cytoskeletal stiffness is primarily determined by actomyosin fibers [106]. Erlach et al. showed that, in comparison to cells cultured on square, and circular micropatterns, the Young's modulus of MSCs was higher on triangular micropatterns [77]. Wang et al. reported that MSC cytoskeletal stiffness was determined by the F‐actin structure, which was affected by the micropatterns controlled cell spreading. When MSCs were cultured on micropatterns with a large spreading area, more stress fibers were generated, resulting in a greater cytoskeletal stiffness. However, different shapes of micro patterns of the same size did not affect cytoskeletal stiffness [92]. Moreover, the size of the micro pattern, combined with the stiffness of the substrate, regulates the stiffness of the cell cortex. Tee et al. showed that when MSCs were cultured on soft substrates, cell cortical stiffness was not affected by the cell adhesion area, but when MSCs were cultured on small‐area micro patterns, the cortical stiffness was not affected by the substrate stiffness [107].

### 3.6 Cell Migration

Cell migration is involved in tumor invasion, embryonic development, immunological processes, and wound healing [108, 109]. Micropatterns have been used to study cell migration. Yao et al. developed micropatterns with microstripes to guide cell adhesion and migration. Cell migration was primarily influenced by the width and arc radius of micro stripes. MSCs cultured on 20‐µm‐wide straight micro stripes migrate faster than the cells cultured on micro stripes with the width of 4, 10, 50, and 100 µm. The speed of MSCs was faster in the counterclockwise direction than in the clockwise direction, while this phenomenon was not observed in NIH3T3, and HeLa cells [80]. Kushiro et al. fabricated combined micro patterns composed of four teardrop micro patterns to study cell migration. The direction of MCF‐10A cell migration was determined by lamellipodial extensions. A closer distance between two teardrop micropatterns could increase the propensity for migration [110].

## 4 Micro patterns as a novel 3D Cell Culture Platform

Micro patterns are beginning to be used as a novel 3D cell culture platform [31], and are generated by coating a matrix (carbohydrates, peptides, and proteins) with a specific shape and size onto planar substrates [33]. Micro patterns not only provide sites for cells to adhere to, but also limit the space available for cell growth [111]. Spatial confinement of cell growth on micro patterns, resulting in the gradual and spontaneous assembly of size‐controllable cell spheroids through cell proliferation, cell‐cell adhesion, and the cell crowding force [27, 30, 112].

**FIGURE 5:**
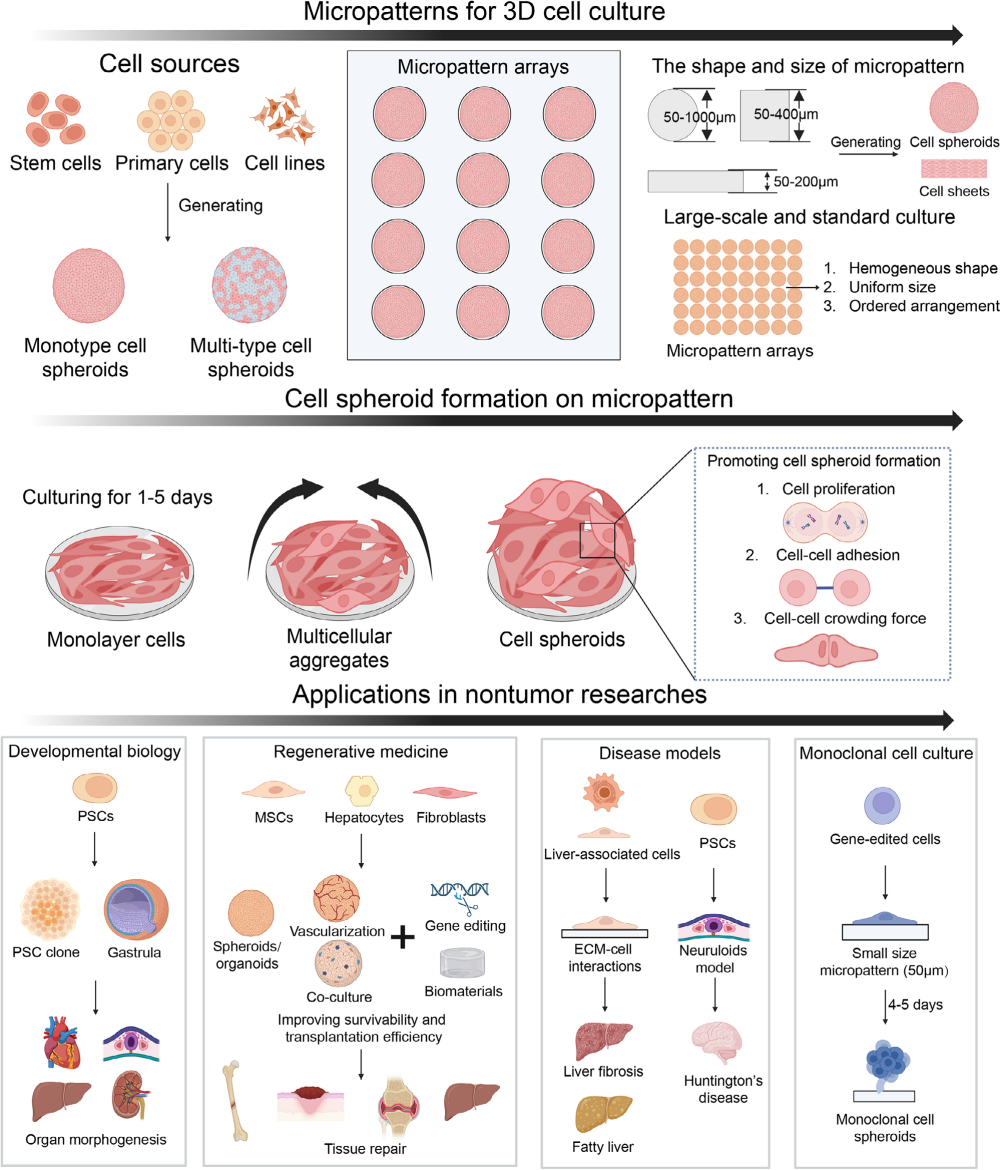
Micro pattern as a novel 3D cell culture platform in non‐tumor researches.

**FIGURE 6:**
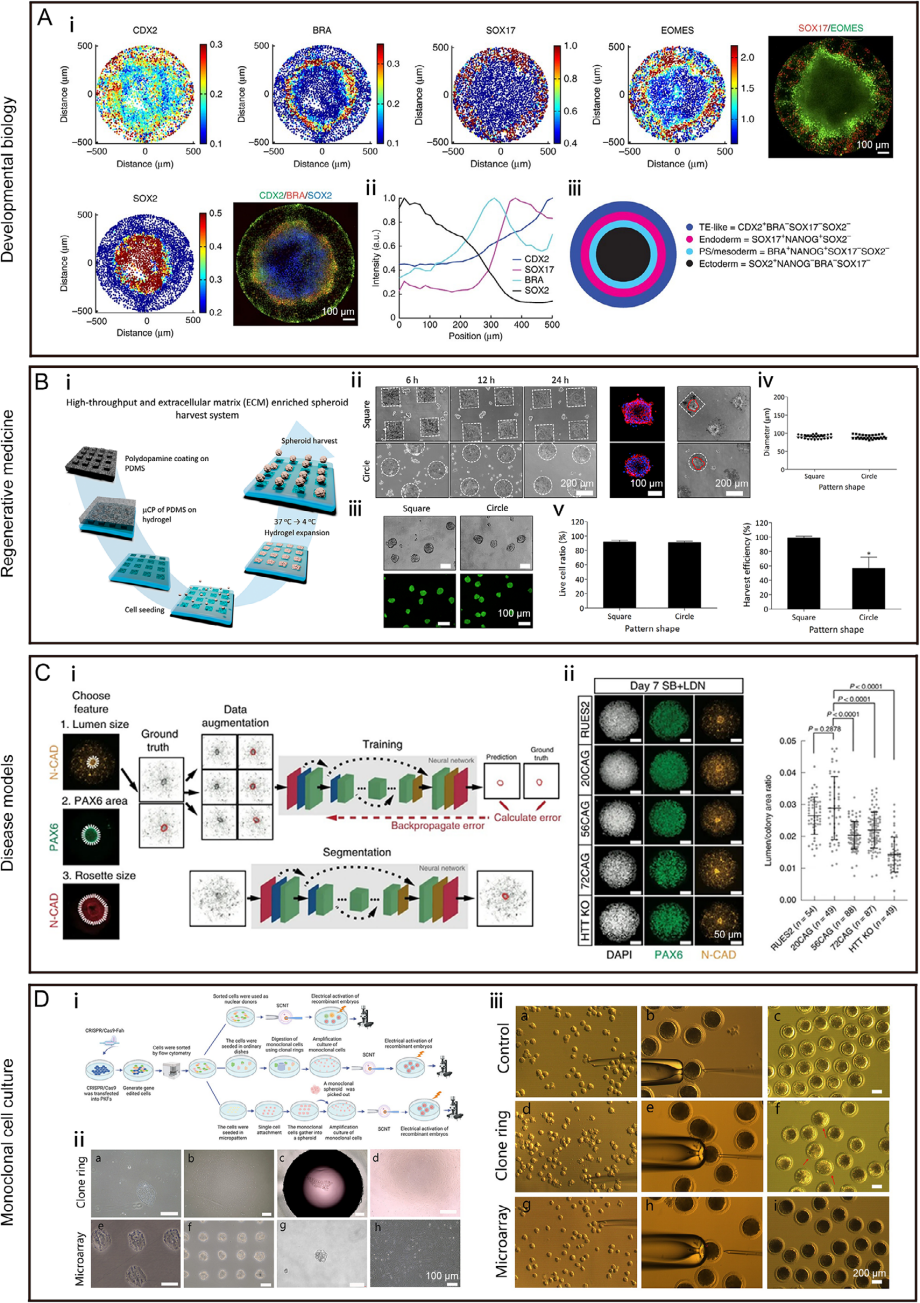
The applications of micro patterns in non‐tumor research, including developmental biology, regenerative medicine, disease models and monoclonal cell culture. (A) Human embryonic stem cells (hESCs) differentiated on micro patterns form self‐organized spatial patterns: (i) immunofluorescence staining of different germ layer markers, when hESCs were seeded on micro patterns, grown overnight and then treated with BMP4 for 42 h; (ii) quantification of the immunofluorescence staining; and (iii) schematic of the results of 42 h of BMP4 treatment in micro patterned culture. Reproduced with permission [115]. Copyright 2014, Springer Nature; (B) MSCs cultured on micro patterns form cell spheroids fore regenerative medicine: (i) schematic diagram of the process of harvesting ECM‐enriched stem cell spheroids using a thermally expandable hydrogel with cell adhesive micro patterns; (ii) MSCs micro layers, f‐actin staining of MSCs micro layers and the harvesting process of MSCs spheroids on the micropatterned hydrogels; (iii) live/dead staining of harvested MSCs spheroids; (iv) diameters; and (v) cell viability and harvesting efficiency of MSCs spheroids according to the shape of micro pattern. Reproduced with permission [117]. Copyright 2018, Elsevier; (C) self‐organizing neuruloids model developmental aspects of Huntington's disease on micro patterns: (i) schematic presentation of phenotypic signatures associated with Huntington disease using deep neural networks, three features of interest are segmented and quantified: lumen size, PAX6 area and overall rosette size; and (ii) representative images and quantification of lumen sizes for different Huntington isogenic lines in the rosette formation assay. Reproduced with permission [118]. Copyright 2019, Springer Nature; and (D) evaluation of the effect of producing recombinant embryos from monoclones obtained by micropatterns: (i) schematic presentation of producing recombinant embryos from monoclones obtained by cloning rings or micro patterns; (ii) monoclonal cells were obtained by cloning rings and micro patterns; and (iii) monoclonal cells were further used to the construction and electrical activation of recombinant embryos. Reproduced under the terms of the CC‐BY‐NC‐ND 4.0 [116]. Copyright 2022, The Authors, published by American Chemical Society.

Although various 3D cell culture technologies (Table 2) have been used to prepare cell spheroids, there are still some limitations, including cost, efficiency, culture time, throughput, uniformity, repeatability, observation, and analysis [26]. Orderly arrayed micro patterns enable large‐scale culture production, control cell spheroid arrangement, and increase throughput [27, 29]. In addition, cell spheroids of controllable size and orderly arrangement are conducive to the observation and analysis, suitable for high‐throughput drug screening and beneficial for improving the authenticity and repeatability of experiments [30, 113]. Micro patterns, as platforms for 3D cell culture, have been widely used to culture stem cells, primary cells, cell lines and tumor cells for the generation of cell spheroids, developmental biology, regenerative medicine, disease models, monoclonal cell culture, tumor research and pharmaceutical research, and development [34, 114, 115, 116]. This section mainly describes and discusses the applications of micro patterns in non‐tumor research (Figures 5 and 6).

### 4.1 Developmental Biology

To study organogenesis, model diseases, and advanced cell therapy, stem cells have been used to form organoids or cell spheroids [17, 45, 119]. For example, during embryogenesis, the gut tube can further differentiate into the lung, oesophagus, stomach, and intestine, which is difficult to recapitulate in vitro because the generation of gut spheroids through traditional monolayer‐based culture is inefficient and unstable [120, 121]. Lin et al. demonstrated that compared with a monolayer‐based culture, a micro patterned gut spheroid generator clearly enhanced the generation of gut spheroids by mechanically improving tissue morphogenesis. The yield of gut spheroids was improved 14‐fold. Moreover, the shape and size of the micro patterns had no effect on the biogenesis of gut spheroids. This biogenesis was related to cell multilayering and crowding, which were caused by mechanical forces [112]. During the process of embryonic development, embryonic cells are differentiated and allocated into three germ layers (the endoderm, mesoderm, and ectoderm) in an ordered spatial sequence, which is difficult to recapitulate in vitro by classical cell culture methods [122]. Warmflash et al. showed that embryonic stem cell (ESC) colonies cultured on circular micro patterns could reflect different germ layers, from outside to inside, as follows: the trophectoderm, endoderm, mesoderm and ectoderm [115]. Furthermore, micro patterns, as a research tool, have been widely used to understand neural, cardiac, hepatic, and renal development [123, 124, 125, 126]. Karzbrun et al. developed micro pattern chips that recreate neural tube folding by precisely controlling 3D cell‐fate patterns and organ shapes. After neural‐inducing differentiation, a millimeter‐long neural tube was generated through the folding of neural ectoderm, which was similar to the development of the neural tube in vivo [127]. Moreover, Seo et al. used micro patterns to generate spinal cord organoids with a self‐organized dorsoventral polarity, which could be used to understand cell patterning and axis formation during neural development [128]. Xu et al. demonstrated that micro pattern could be used to generate liver organoids to overcome the drawbacks of the Matrigel dome method, such as the heterogeneity of morphology, size, and maturity and random arrangement. These micropatterned liver organoids recapitulated several key characteristics of liver development and enabled in situ staining and imaging [129]. Micro patterns provides us with an approach that allows us to study positive and negative regulatory mechanisms of cell fate and behavior at the same time. Billet et al. used micro patterns with both attractive cue Collagen XV‐B, and repulsive cue Tenascin C to mimic the heterogeneous ECM and successfully induced directional motor axon growth [130].

### 4.2 Regenerative Medicine

Micro patterns have been used to culture MSCs, hepatocyte, and fibroblast spheroids to promote cell functions and in regenerative medicine [131, 132, 133]. In the micro pattern culture platform, large‐scale and standardized culture of vascularized MSC spheroids can be achieved [117, 134]. For example, hepatocyte spheroids (cocultured with fibroblasts that were modified by gene editing to interact with the ECM) have been developed [132, 135, 136]. Fibroblasts can form micro tissues with a controlled size, high viability, abundant ECM, and junction proteins or stem cell‐like spheroids [137, 138]. In addition, the editable nature of the tissue structure provided by micro pattern technology allows us to fabricate fine structures composed of different types of cells. For example, a two‐well culture chamber with micro patterned lanes of ECM is used to culture hESC‐derived skeletal myocytes and primary tenocytes, and induce the formation of myotendinous junction‐like structures [139].

#### 4.2.1 MSCs

With their extensive sources (bone, adipose tissue, and umbilical veins), self‐renewal ability and differentiation potential, MSCs are considered promising cell sources for stem cell‐based therapy [15, 119, 140]. In 2D culture, MSCs can gradually lose cell stemness and the secretory ability for significant molecules and factors [141]. 3D stem cell spheroids in which stem cells can interact with stromal cells and the ECM to rebuild the niche microenvironment can effectively address these problems [10]. Wang et al. developed a micro pattern system to generate precisely uniform MSC spheroids that possessed greater differentiation potential, thereby achieving the integration of spheroid culture and differentiation. By up regulating adipogenesis‐ and osteogenesis‐related genes and downregulating MSCs self‐renewal‐related genes, these systems could tremendously improve the differentiation efficiency [131]. Based on micropatterns, Yanagihara et al. further proposed combining MSCs spheroids with gene transfection to enhance cellular functions and regulate cell fate. MSC spheroids generated in a 100 µm micro pattern array were transfected with a plasmid encoding runt‐related transcription factor 2 (Runx2) to promote bone regeneration, which improved the efficiency of osteogenic differentiation in vitro. Furthermore, when transplanted into rats with bone defects, MSC spheroids could more rapidly migrate into the bone defect region and showed higher recovery of bone defects than 2D cultured MSCs [142]. Li et al. discovered that triangular and rectangular micro patterns could enhance the paracrine function of MSCs more effectively than circular and square micro patterns by promoting the nuclear import of YAP, which in turn led to the acceleration of wound repair in a wounded rat model [91].

To further efficiently fabricate and quickly harvest MSC spheroids, Lee et al. formed micro patterns on the surface of thermo sensitive hydrogels and showed that human turbinate MSCs could assemble into micro scale cell sheets. As the temperature decreased from 37°C to 4°C, the MSC sheets were released from the surface of the thermo sensitive hydrogel, after which cells self‐assembled into spheroids with potentiated cell‐cell and cell‐ECM interactions and consistent sizes. Compared with circular micro patterns, square micro patterns were more beneficial for the self‐assembly of MSC sheets. Micro pattern‐generated MSC spheroids showed greater expression of ECM proteins, stemness markers and greater osteogenic, chondrogenic and adipogenic differentiation efficiencies than the spheroids that generated by the microwell method [117]. Moreover, Byun et al. further proved that the size of square micro patterns on the thermo sensitive hydrogel could determine the size of MSC spheroids. The origin of MSCs (turbinate, adipose or bone marrow tissue) did not affect their self‐assembly on square micro patterns, and the stemness of MSCs was not related to the spheroid size. During the process of self‐assembly, E‐cadherin expression increased and N‐cadherin expression decreased in MSC spheroids, which was related to a key mechanism of MSCs self‐assembly. MSC spheroids showed a high viability rate (up to 90%) after long‐term suspension culture (7 days), syringe injection, and cryopreservation, providing a good basis for the clinical application of MSCs [143].

Using a thermo sensitive hydrogel coated with micro patterns, Kim et al. developed more complex vascularized MSCs spheroids through coculture with human umbilical vein endothelial cells (HUVECs). Cell seeding by serial (first seeding MSCs, then seeding HUVECs) or mixed (seeding MSCs mixed with HUVECs) method could affect the vascularized structure of cell spheroids and the degree of vascularization. When using serial seeding, the core of the cell spheroids was formed by MSCs, and HUVECs were distributed on the periphery. However, mixed seeding resulted in a non‐polarized distribution of MSCs and HUVECs within the spheroids. Furthermore, after three days of culture, the outer HUVECs fused to the core of the spheroids, and the vascularization in serially seeded spheroids was greater than that in mixed‐seeded spheroids [134].

Generally, the combination of micro pattern technology to generate MSCs and vascularized MSC spheroids provides a new perspective for the fabrication of 3D complex micro tissues, MSC treatment and clinical transformation.

#### 4.2.2 Hepatocytes

In 2D cell culture, some primary cells are difficult to survive, and cell characteristics tend to weaken or change during the passage process [3]. For example, primary hepatocytes are difficult to culture and to passage, and they can easily differentiate and lose hepatic functions, which can be improved through spheroid or organoid culture [56]. Kojima et al. showed that fetal mouse liver cells, which were defined as a population of immature cells, formed spheroids when cultured on micro patterns. Moreover, after being cocultured with nonparenchymal cells, the degree of maturity and hepatic function (albumin secretion) of fetal mouse liver cells was significantly increased [132]. Miyamoto et al. reported that bovine carotid epithelial HH cells could form a layer of feeder cells on a micro pattern, which supported primary hepatocytes in forming spheroids with strong hepatic function [144]. Liu et al. demonstrated that primary hepatocytes cocultured with 3T3 fibroblasts had much higher albumin, urea, and P450 expression on micro patterned fibers than on random fibres [145]. Being less expensive and having more reproducible traits, HepaRG cells can be considered a hepatocyte replacement [146]. Ware et al. showed that coculture of HepaRG cells with 3T3 cells on micro patterns increased albumin secretion and promoted more stable cytochrome P450 activity than monolayer or random culture. This coculture system displayed high sensitivity for drug evaluation [147].

To improve cellular functions and paracrine effects, Endo et al. developed a micropattern culture system combined with a nonviral vector (a polyplex nanomicelle) to produce genetically modified cell spheroids. Using this platform, primary hepatocyte spheroids that were transfected with Gaussia luciferase and were able to continuously express albumin and luciferase for up to one month [148]. Furthermore, Uchida et al. confirmed the feasibility of combining micropatterns with polyplex nanomicelles to generate hepatocyte spheroids via genetic modification for cell transplantation therapy. Compared to hepatocytes derived from monolayer culture, the hepatocyte spheroids that transfected with a plasmid encoding the erythropoietin gene exhibited consistently higher erythropoietin expression in vivo for more than one month and had greater haematopoietic effects [135]. This genetically modified spheroid system could be applied not only to primary hepatocytes but also to a variety of cell types, including MSCs [149].

Monckton et al. utilized micro patterns to assess primary hepatocyte attachment and functions, which may be related to ECM protein species and matrix stiffness. High stiffness (25 kPa) was more conducive to hepatocyte attachment than low stiffness (1 kPa). In addition, after 14 days of culture, albumin and HNF4α levels were higher in the 25‐kPa stiffness group. ECM protein species could modulate hepatocyte functions at 1 or 25 kPa stiffness. Collagen type IV combined with hyaluronic acid induced greater CYP3A4 expression at 1 kPa, while collagen type V induced greater HNF4α expression at 25 kPa over a period of 14 days [136].

#### 4.2.3 Fibroblasts

Micro patterns have also been used to culture fibroblasts to generate micro tissues. Yu et al. developed multi‐layer cell‐collagen constructs via micro pattern to treat bone injuries. Aligned collagen cultured MC3T3‐E1 cells were used to generate single‐layer cell‐collagen constructs, which were stacked in multiple layers to form biomimetic multi‐layer cell‐collagen constructs. Compared to either cell alignment or mechanical conditioning used in isolation, these constructs, which had both an aligned arrangement of cell‐collogen and mechanical conditioning, showed the highest rate of osteogenic differentiation in vitro and the highest bone formation capability in vivo [150]. As described above, micro patterned thermo sensitive hydrogels can be used to culture fibroblasts. Lee et al. reported that linear micro patterns were used to culture human dermal fibroblasts (HDFBs) to generate micro tissues with a controlled size, high viability, and abundant ECM and junction proteins. When subcutaneously transplanted into mice, micro tissues could be retained in vivo up to 7 days and became linear through migration. These scaffold‐free micro tissues could be expected to solve the problem of cell retention in single‐cell transplantation [133]. These micro patterned thermo sensitive hydrogels could be used to culture MSC spheroids and HDFB microtissues [117, 133]. However, the mechanism of spheroid or micro tissue self‐assembly is complex and worth exploring. Kim et al. reported that the number of cells and the shape of micro patterns govern spheroid self‐assembly. A large number of cells was beneficial for improving the tugging force, which could induce spheroid self‐assembly. When circular micropatterns were used, the percentage of rat spheroids generated decreased to 60%. The process of self‐assembly was defined as the mode of outside‐in assembly through mechanically induced interactions between the hydrogel and cells [137]. Roy et al. demonstrated that fibroblasts cultured on micro patterns could form stem cell‐like spheroids, which had important implications for regenerative medicine [138].

#### 4.2.4 Other Cells

A well‐organized vascular network is essential for the functional architecture of tissues and organs. Micro patterned gelatin methacrylate (GelMA) hydrogels (50–150 µm height) were used to direct multicellular morphogenesis of endothelial cells in 3D to assemble into endothelial cord structures with organized actin fibers and circular/elliptical cross‐sections, which are a precursor to tubulogenesis [151]. Under shear flow, micro pattern alignment parallel to the flow direction could increase the flow enhanced cell‐ECM adhesion of HUVECs, suggesting the importance of topographical and shear flow cues in the design of functional vascular grafts [152]. This spatial confinement induced cellular elongation of micro vascular endothelial cells by micro patterning differs from the mechanical response to shear forces or topography [153]. Li et al. reported that parallel micro‐stripes of high molecular weight hyaluronic acid on the titanium surface regulated the anticoagulant factors secretion of endothelial cells [154]. In addition to endothelial cells, cardiomyocytes can also respond to isotropic biomimetic micro patterns based on embryonic chick myocardium (2 or 20 µm wide) to modulate maturation and intercellular interaction [155]. Appropriate micro patterned structures may also be involved in the regulation of myoblast cellular organization and the cell fusion process. Myoblasts on micro patterned PDMS films showed a well‐organized cytoskeleton and enhanced myotube formation [156]. By combining synNotch receptor technology with micro patterns, Garibyan et al. were able to spatially control differentiation in multicellular constructs and engineer aligned muscle tissue [157].

### 4.3 Disease Models

Micro patterns are widely used to construct in vitro models of certain diseases. For instance, liver sinusoidal endothelial cells (LSECs) play an important role in the progression of liver fibrosis [158]. Brougham‐Cook et al. reported that LSECs responded to ECM proteins, matrix stiffness, and soluble factors in liver fibrosis based on micro patterns. LYVE‐1 and CD31 expression was greater at a stiffness of 1 kPa, while E‐cadherin expression were greater at a stiffness of 25 kPa. LYVE‐1, E‐cadherin, and CD31 expression was unregulated by collagen type IV [159]. Moreover, Jain et al. cultured primary hepatic stellate cells (HSCs) on micro patterns with different stiffness's and matrices to understand the relationship between ECM deposition and remodeling in HSCs during liver fibrosis in nonalcoholic fatty liver disease. Compared with those in the 25 kPa group, chromatin accessibility was higher, and fibrosis‐related genes were unregulated in the 1 kPa group [160]. For understanding changes in the ECM and cellular topology during the development of idiopathic pulmonary fibrosis, Zhu et al. designed the rectangle and square micro patterns to induce aligned (anisotropic) or random (isotropic) arrangement of MRC‐5 lung fibroblasts to investigate the effects of cellular topology on cellular fates. Compared with anisotropic MRC‐5 cells, isotropic MRC‐5 cells showed changes of the cytoskeleton and developed a highly invasive phenotype [161]. Meanwhile, the ECM and cellular topology also play an important role in myocardial infarction (MI). Bugg et al. discovered that myofibroblasts were mainly located in anisotropic ECM fibers rather than in isotropic ECM fibers after MI. Aligned micro patterns (anisotropy) led to elongation and a higher differentiation rate of α‐smooth muscle actin (α‐SMA)+ and periostin (POSTN) + MFs than randomly oriented micro patterns (isotropy) in vitro, and then aligned micro patterns promoted the differentiation of myofibroblasts through mechanical activation of p38‐YAP‐transcriptional enhanced associate domain signaling [162].

In understanding human genetic diseases such as Huntington's disease, in vitro models rarely reflect morphogenesis, while animal models often face homozygous lethality [118, 163]. Haremaki et al. developed micropattern technology to generate neuruloids containing neural progenitors, neural crest cells, sensory placodes, and the epidermis to recapitulate early human neurodevelopment (neurulation) through dual SMAD inhibition and bone morphogenic protein 4 stimulation. During the self‐organization of neuruloids, the pulse of pSMAD1 could induce epidermis formation at the edge and was accompanied by central neural fates that specified the neural crest and placodes and were regulated by fibroblast growth factor and Wnt. Through the use of isogenic ESCs from Huntington's disease patients to fabricate neuruloids, early morphogenetic defects were connected with the impairment of the actin‐mediated tissue organization mechanism [118].

### 4.4 Monoclonal Cell Culture

Compared to classical monoclonal cell culture methods (the limit dilution method or clone cycle digestion method), micro patterns can provide the ECM microenvironment for cell population growth and reduce the culture time [116]. Zhang et al. showed that gene‐edited porcine iliac artery endothelial cells (PIECs) could grow into cell spheroids on micropatterns (circular patterns of 50 µm in diameter) within four days, which brought convenience for harvesting monoclonal cells [164]. In addition, Gao et al. utilized small micro patterns (circular patterns of 50 µm in diameter) to obtain monoclonal primary cells. After five days, gene‐edited renal fibroblasts were able to form monoclonal cell spheroids, which improved the efficiency of obtaining gene‐edited monoclonal cells and reduced cell damage [116].

### 4.5 Other Applications

Kutsuzawa et al. developed a micro pattern coculture system to generate Chinese hamster ovary cell spheroids using bovine aortic endothelial cells or 3T3 cells as feeder cells to produce recombinant proteins. Compared with normal spheroid culture, this system improved protein expression up to 3‐fold [165]. Otsuka et al. developed a non‐invasive micro pattern monitoring system to study cellular uptake kinetics. Changes in the extracellular potential were found to be affected by interactions between ECM proteins and substrates in the cell membrane. On micro patterns, functional changes in the cell membrane of chondrocyte spheroids could be detected by using a chondrocyte‐based field‐effect transistor [166].

## 5 Application of Micro Patterns in Tumor Research

**FIGURE 7:**
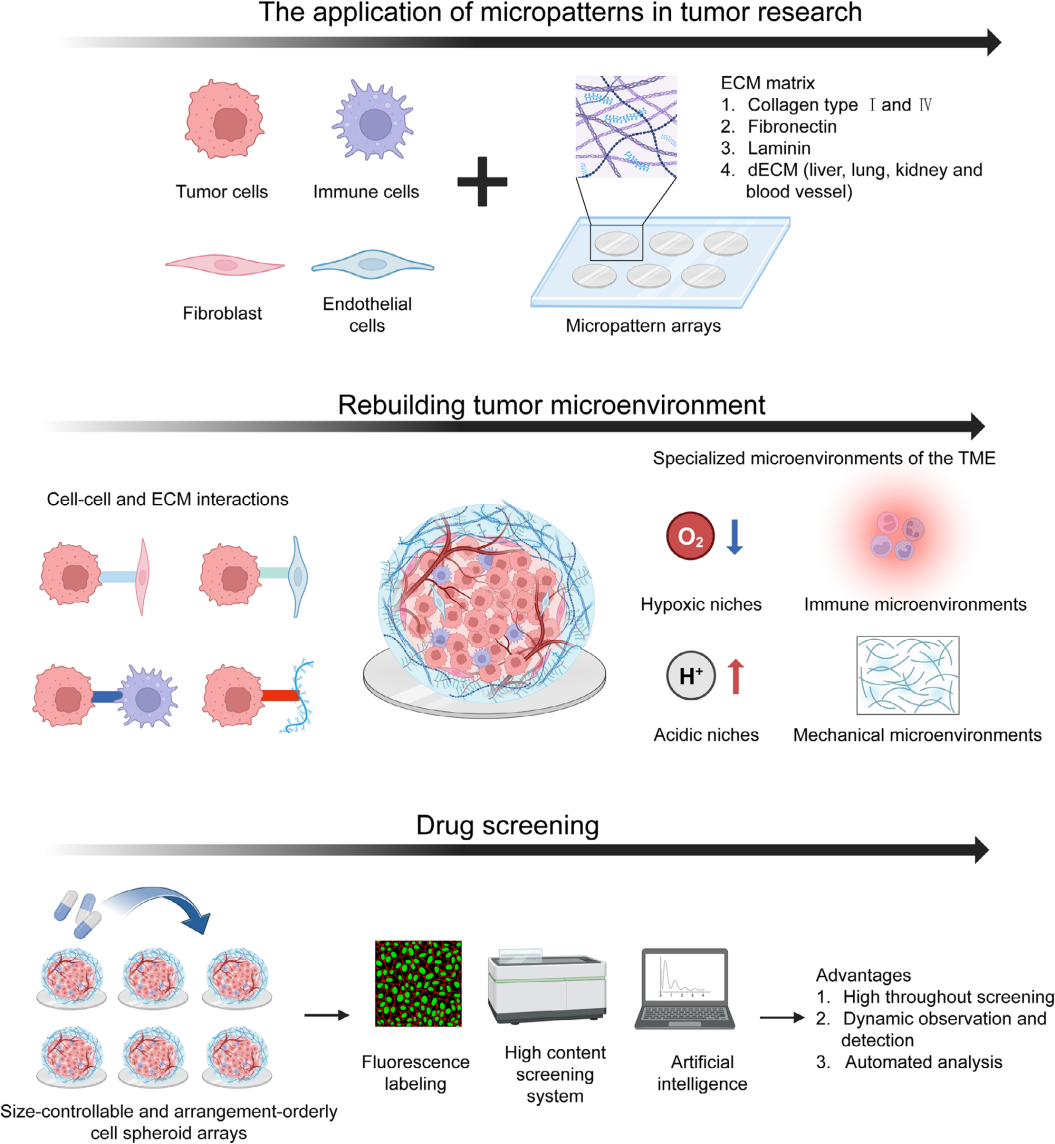
Schematic illustration of the perspective application of micro patterns in tumor research.

**FIGURE 8:**
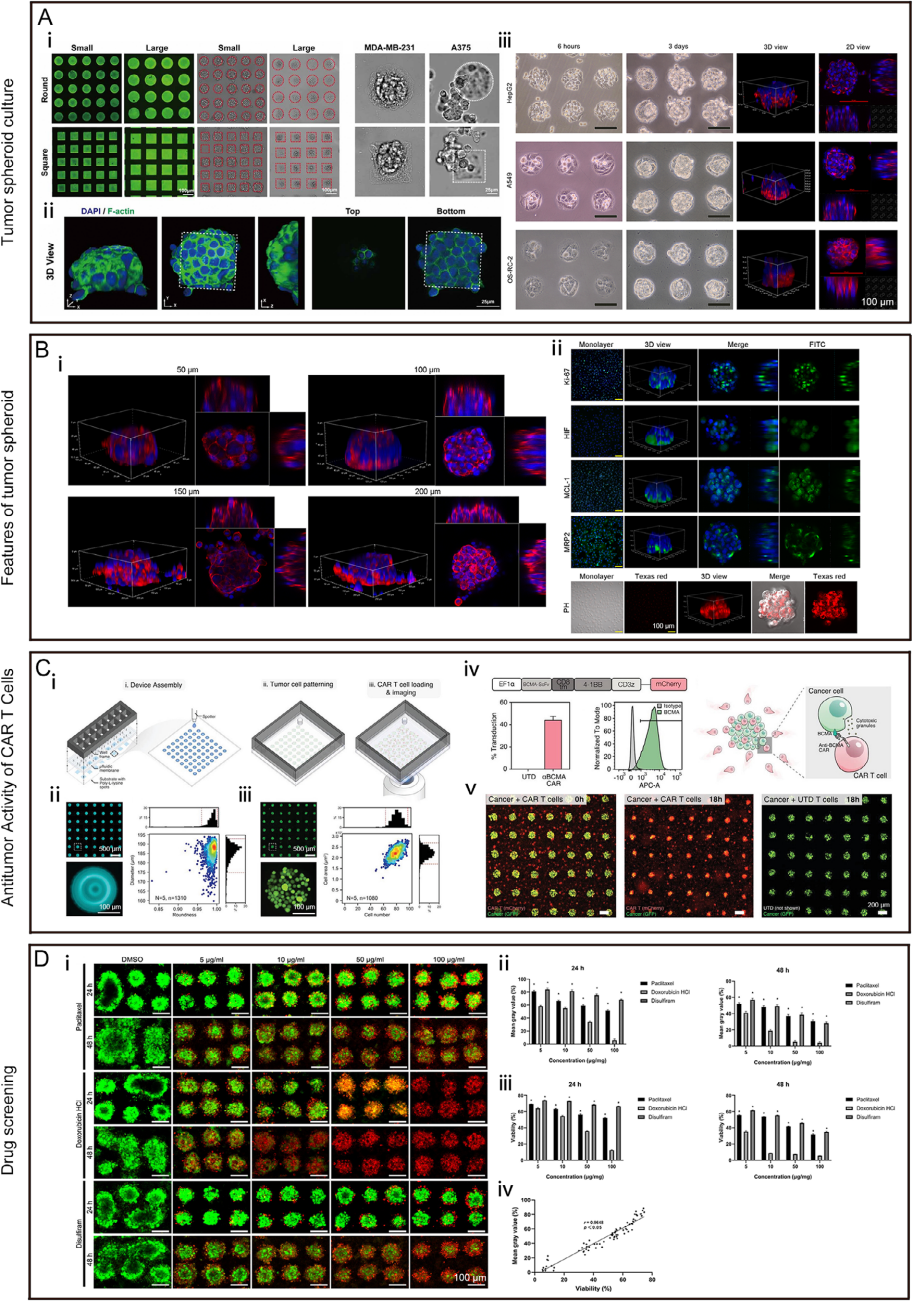
The applications of micro patterns in tumor research. (A) Micro patterns used as the platform for tumor spheroid culture: (i) HeLa, MDA‐MB‐231, and A375 cells cultured on the fibronectin inked micro pattern array; (ii) 3D view of HeLa spheroid in single square micro pattern. Reproduced with permission [30]. Copyright 2021, Institute of Physics; and (iii) Tissue‐specific micro pattern array chips for 3D cell culture (HepG2 cells, A549 and OS‐RC‐2 cells). Reproduced under terms of the CC‐BY license [170]. Copyright 2023, The Authors, published by Elsevier; (B) features of A549 spheroids cultured on micro patterns: (i) 3D view of A549 spheroids in micro pattern with different diameters (50, 100, 150, and 200 µm); (ii) immunofluorescence staining of Ki67 (proliferation marker), HIF‐1α (hypoxia marker), MCL‐1 (anti‐apoptosis marker), MRP2 (drug resistance), cytosolic pH in A549 cell spheroids (formation on 100 µm micro pattern), and monolayers. Reproduced with permission [29]. Copyright 2022, Elsevier; (C) micro patterned tumor arrays (MiTA) for the quantification of CAR T cell killing: (i) schematic illustrations showing the assembly of the 16 well device, zoom‐in of one well during the printing of the 64 spots, tumor‐cell patterning in the wells, and subsequent CAR T cell loading and imaging; (ii) fluorescent microscopic images of micro patterns and a heat‐scattered plot of the diameter versus roundness of micro patterns; (iii) fluorescent microscopic images of BCMA spheroids and a heat‐scattered plot of cell number versus cell spheroids area; (iv) schematics of second‐generation anti‐BCMA chimeric antigen receptor (CAR) construct and BCMA–anti‐BCMA–CAR interactions mediate tumor cells (green) recognition and killing by CAR T cells (red); and (v) fluorescent microscopic images showing the snapshots of the interaction of CAR T cells (red) and tumor cell islands (green) at 0 and (ii) 18 h. Reproduced under terms of the CC‐BY license [171]. Copyright 2019, The Authors, published by John Wiley and Sons; and (D) HepG2 spheroids cultured on micro pattern for anticancer drug screening: (i) live/dead staining of HepG2 spheroids with various concentrations (5−100 µg mL^−1^) of paclitaxel, doxorubicin HCl, and disulfiram. Quantitatively analyzing the efficacy of an anticancer drug by (ii) mean gray value (according to Live/Dead staining); (iii) CCK‐8; and (iv) correlation between mean gray value and viability detected by CCK‐8. Reproduced under the terms of the CC‐BY‐NC‐ND 4.0 [27]. Copyright 2022, The Authors, published by American Chemical Society.

During tumor progression, the complex process of tumorigenesis, including initiation, progression and metastasis, is under the control and regulation of the TME [167, 168]. Tumor spheroid models can mimic the multifaceted TME to better understand and study the complex versatility of tumors, which will facilitate the development of anti‐tumor therapies and drugs [18, 39]. Currently, the use of tumor spheroid models in combination with bio fabrication represents a significant advance in the characterization of the complex TME and drug development [40, 169]. Micro pattern array chips not only support the culture of tumor spheroids, but also reconstitute the TME in vitro and contribute to the accuracy, stabilization and automation of drug screening (Figures 7 and 8).

### 5.1 Tumor Spheroid Culture

Micro patterns can be used to effectively culture multicellular spheroids in in vitro as tumor models with 3D structures to understand tumor growth and monitor the dynamic progression of tumors in real time [27, 171]. Tamura et al. fabricated micro patterns of collagen to culture HepG2 spheroids that showed ECM deposition and survived for up to 2 weeks. HepG2 spheroids larger than 180 µm in diameter showed necrosis in the spheroid core. As the spheroid diameter increased, albumin secretion improved [172]. For better observation and analysis of tumor spheroids, Lee et al. developed micropatterns consisting of double‐layered bare polystyrene (BP) nanofibers through electro spinning and lithography. The top layer of the nanofibers supported HepG2 spheroid culture and controlled its size, while the bottom layer of the nanofibers detected the secretion of the HepG2 spheroids, advancing the progress of spheroid culture miniaturization and detection [173]. Moreover, Liu et al. showed that amine‐rich micro patterns supported the culture of MCF‐7 spheroids for 24–48 h and maintained their morphology for 14 days. Using this platform, the ability of transferrin gold nanoparticles to effectively combine with MCF‐7 spheroids was verified and observed. These results facilitated the further advancement of on‐chip assays for the study of tumors [174]. To quickly harvest tumor spheroids, Jiang et al. cultured pancreatic tumor spheroids for 5 days and reported that spheroids with 99% viability could be generated and released from micro patterns by changing the temperature. Micropatterns were fabricated from a two‐layer PDEGMA matrix with different thicknesses (5 and 40 nm), with cell adhesion relying on fibronectin. The 40‐nm PDEGMA matrix could not adhere to fibronectin at 25 or 37°C, while the 5‐nm PDEGMA matrix could not adhere to fibronectin at 25°C but could at 37°C. As the temperature dropped, the tumor spheroids were easily released and harvested [175].

### 5.2 Rebuilding the TME

The TME is defined as the dynamic ecosystem that is composed of tumor cells, TME‐associated cells, and the ECM that determines tumor heterogeneity, evolution, progression, metastasis and resistance [176, 177]. Studies of the cellular and ECM components of the TME and on the specialized microenvironment of the TME have contributed to the discovery of potential therapeutic targets and stimulated the development of treatments targeting the TME [168]. Recently, micro patterns have been used to study the interactions between tumor cells, TME‐associated cells, and the ECM and to rebuild hypoxic niches, acidic niches, immune microenvironments and mechanical microenvironments.

#### 5.2.1 TME

The TME is composed of multiple types of non‐tumor cells and the ECM. The dominant cell types in the TME are immune cells, stromal cells and tumor endothelial cells (TECs). According to its characteristics and functions, the TME is classified into a hypoxic niche, a metabolic microenvironment, an acidic niche, an innervated niche, an immune microenvironment, and a mechanical microenvironment [167, 178].

Immune cells, including macrophages, neutrophils, dendritic cells, natural killer (NK) cells, and T cells, determine the tumor immune microenvironment and exhibit tumor‐promoting, or anti‐tumor effects [179, 180]. Stromal cells, including fibroblasts and pericytes, are involved in tumor growth and metastasis [178]. Cancer‐associated fibroblasts (CAFs) can secrete and model the ECM to adjust its mechanical properties and regulate tumor angiogenesis and metastasis, as well as immune cell behavior [181, 182]. Pericytes have gradually attracted increasing attention for their tumor immunomodulatory ability [183]. TECs contribute to the formation of blood vessel formation and are involved in tumor angiogenesis, progression, metastasis, and resistance [184]. In addition, the ECM is a dynamic network that includes collagens, glycoproteins, proteoglycans, chemokine's, cytokines and growth factors. The ECM accounts for approximately 60% of the mass of solid tumors and provides a variable matrix with various elasticity and stiffness that regulates tumor growth and metastasis [168, 178, 185].

#### 5.2.2 Interactions Between Tumor Cells, TME‐Associated Cells, and the ECM

Micropattern array chips can serve as a platform to study the interactions between tumor cells, TME‐associated cells and the ECM. Micro patterns have been used to study interactions between tumor cells and fibroblasts and between tumor cells and endothelial cells. Kikuchi et al. constructed a micro pattern coculture system to coculture HepG2 cells with 3T3 cells. HepG2 cells were first seeded into micro patterns for 3 days to form spheroids, after which 3T3 cells were seeded. As the culture time increased, 3T3 cells gradually invaded the HepG2 spheroids. After coculture with 3T3 cells, the morphology of the HepG2 spheroids was stabler and the expression of CYP3A4, CYP1A2, and albumin improved [186]. Li et al. also cultured HeLa cells on micro patterns to form spheroids, after which 3T3 cells mixed with the GelMA hydrogel were seeded into the micro patterns to mimic interactions between fibroblasts and the ECM [30]. Yoshimoto et al. demonstrated that bovine aortic endothelial cells used as feeder cells supported the growth of human hepatoma cancer cells (FLC‐4) [187]. As described above, using micropatterns as a culture system, MSCs were cocultured with HUVECs to establish vascularization, which proved that micro patterns might rebuild the vascularization of tumor spheroids in vitro [134].

Hadavi et al. used micro patterns as a tool to study the effects of ECM proteins (fibronectin, collagen type IV, and laminin) on insulinoma cell spheroids. In contrast to laminin, fibronectin, and collagen type IV facilitated insulin secretion. However, laminin was more beneficial for the growth of spheroid than fibronectin and collagen type IV [188].

#### 5.2.3 Hypoxic and Acidic Niches

The overgrowth and limited vascularization of tumors lead to an insufficient oxygen supply, and the induced hypoxic niche changes tumor metabolism [189]. The hypoxic niche is related to tumor progression, poor prognosis, and resistance [167, 168]. The acidic niche is caused by hypoxia and the accumulation of lactate [190]. Initially, in the early stage, the acidic niche induces tumor apoptosis; however, a persistent acidic microenvironment promotes tumor adaptation and hinders therapy [176, 191, 192].

Ma et al. developed a micropattern system to culture hypoxic U87 glioma spheroids. On the micropatterns, U87 cells could form spheroids, which were transfected with hypoxia‐inducible factors (HIFs). U87 glioma spheroids overexpressing HIFs exhibited increased invasion and migration through epithelial‐mesenchymal transition (EMT) in the matrix [193]. Miyamoto et al. evaluated the effect of the gap distance of micro patterns on the growth, function, and hypoxic niche of HepG2 spheroids. The albumin secretion by HepG2 spheroids was minimally influenced by the gap distance of the micro patterns. When the distance between the gaps of the micro patterns was less than 1000µm, HepG2 spheroid growth was inhibited, and anaerobic metabolism was improved. These effects disappeared when the gap between the micro patterns exceeded 1500 µm [194]. Zhu et al. showed that A549 spheroids cultured on micropatterns had a hypoxic core with higher expression of HIF‐1α than that in monolayer cells and a lower cytosolic pH than that in periphery and monolayer cells, which was similar to primary infantile haemangioma spheroids [29, 113].

#### 5.2.4 Immune Microenvironments

The immune microenvironment is associated with tumor progression, metastasis and treatment. Its major functions regulate immune cells and control chemokine's, cytokines, and growth factors [195, 196, 197].

CAR T‐cell therapy is regarded as the most promising tumor immunotherapy method. During the development and preclinical assessment of CAR T‐cell therapy, appropriate models should be seriously considered [198, 199]. Wang et al. cultured B‐lymphoblast myeloma spheroids on micro patterns, after which CAR‐T cells were randomly seeded into the micro patterns to dynamically simulate CAR‐T cells to be trafficked and kill tumor cells. CAR T cells were first trafficked, then clustered, and finally killed B‐lymphoblast myeloma spheroids, which was observed using real‐time monitoring techniques [171]. This model opened a new door for studying tumor immunology and evaluating cellular immunotherapy.

#### 5.2.5 Mechanical Microenvironments

Apart from biological and chemical cues, the mechanical properties of the microenvironment can also affect the ECM, cell morphology, tumor progression, metastasis and the therapeutic responses [167, 200, 201]. Antmen et al. showed that nuclear deformability could be modulated by the mechanical properties of micro patterns to reflect tumor malignancy levels. Three breast tumor cell lines with different levels of malignancy and invasiveness exhibited different degrees of nuclear deformation. Highly aggressive breast tumor cells, with lower circularity values, were regulated by reduced expression of lamin A/C and nesprin‐2 [202]. Moreover, Wakhloo et al. showed that, based on micropatterns, osteosarcoma nuclear deformation was controlled by actomyosin contractility, vimentin, and nucleocytoskeletal connections but not by A‐type lamin [203]. Li et al. demonstrated that mechanical signals regulated the proliferation and stemness of MDA‐MB‐231 cells. Geometrical confinement generated by micro patterns was shown to promote the expression of Oct4 and Sox2 at the edge of spheroids by up regulating the expression of YAP, which is associated with mechanotransduction [30]. Lin et al. investigated the mechanism by which mechanical cues trigger tumor cell EMT and showed that epithelial tumor cell lines (MCF7, MCF10A, and A549) could autonomously undergo EMT based on the spatial geometric confinement induced by micro patterns, which successfully reproduced the spatial asymmetries of EMT during tumor progression in vitro. In addition, mechanical heterogeneity alone induced the EMT and activated heterogeneous TGF‐β‐SMAD signaling, which was regulated by cell proliferation, migration, and contraction. When cell contraction was suppressed, the EMT process and the activation of TGF‐β/SMAD signaling were inhibited [204].

### 5.3 Drug Screening

Although tumor spheroids can be an ideal model, especially in preclinical drug screening and assessment, drug screening against tumor spheroids is currently limited by two difficulties, namely how to generate standardized tumor spheroids and how to comprehensively evaluate drug screening results [38, 205, 206]. Different methods of generating tumor spheroids affect their various parameters, including the shape, size, 3D density, surface characteristics, and the internal microenvironment. These features of tumor spheroids in turn influence drug penetration, uptake, reaction, half‐life, pharmacokinetics and efficacy [26, 27]. Traditional methods used to evaluate drug efficacy, such as the MTT, MTS, Prussian blue, WST‐8, CCK‐8, and ATP assays, are based on the assessment of cell viability; however, these methods are time‐consuming, and the reagents cannot easily diffuse into tumor spheroids [28]. Moreover, these methods cannot reflect the dynamic process of drug reactions or changes in the tumor spheroid morphology, structure, density, or invasion, although these morphological and structural characteristics have been widely studied [40, 207].

Micropatterns can be useful in standardizing the generation of tumor spheroids. The shape, size, and 3D density of every tumor spheroid and the stability and reproducibility of each batch of tumor spheroids can be controlled by micro patterning [29, 170]. On the other hand, since tumor spheroids are controlled at the same spatial level by micropatterns, micropatterns combined with 3D imaging systems (confocal imaging, high‐content imaging, and multiphoton microscopy) and fluorescent dye and probe methods can dynamically capture the morphology and motility of tumor spheroids and can be combined with artificial intelligence (AI) to perform automated high‐throughput analysis [29, 208, 209, 210]. Recently, a micropatterned chip was fabricated and used to quantify the metastatic potential of cancer cells more sensitively without chemoattractants or microfluidics [211]. Using a SPherSPheroid SPreading on grids analysis platform, gridded micro patterns were used to mimic the networks of the brain vasculature to study the relationship between cell motility modes and mechanical properties, formin content, or substrate chemistry of glioblastioma cells [212]. As discussed above, patterned tumor spheroids mimic, and reflect the TME, which more dependably reflects the drug efficacy. Micro patterns have unique advantages in the application of tumor spheroids for drug screening.

#### 5.3.1 Tumor Cell Lines

To date, several tumor cell lines (HeLa, HepG2, A549, and H1299) and primary CD31+ haemangioma endothelial cells (HemECs) can form tumor spheroids on micro patterns and are used for drug screening. Li et al. cultured HeLa spheroids on micropatterns to evaluate drug efficacy through a calcein‐AM/PI staining cell viability assay [30]. Zhu et al. reported micro patterns that were fabricated by using a decellularized liver ECM to provide a liver tissue‐specific matrix to support size‐controllable and ordered HepG2 spheroid cultures. The decellularized liver ECM was beneficial for improving HepG2 cell adhesion, proliferation and function. Combined with fluorescence staining and confocal microscopy, HepG2 spheroid culture and drug screening were performed on one micro pattern chip. With a dense 3D structure, the surface cells of the spheroids died first, followed by the inner cells. This phenomenon could effectively reflect the authenticity of drug efficacy [27]. Moreover, Zhu et al. generated lung‐derived decellularized ECM (dECM) micro patterns for culturing A549 spheroids with specific features. For example, Ki‐67 and MRP2 were mainly expressed in the periphery of the spheroids, and HIF‐1α and MCL‐1 were mainly expressed in the core of the spheroids, while Ki‐67, HIF‐1α, and MCL‐1 were randomly expressed in monolayer cells. The A549 spheroids exhibited abundant cell‐cell interactions and acidic cores. These specific features explained the difference in the drug screening results between spheroids and monolayer cells and mimicked drug efficacy in vivo [29].

#### 5.3.2 Primary Tumor Cells

Primary tumor cells can truly reflect drug efficacy but often show cell variation and loss of heterogeneity [59]. Li et al. demonstrated that vascular‐specific dECM micro patterns supported the features of primary CD31+ HemECs. Compared with that in 2D culture, the expression of genes related to stem cell pluripotency, the renin–angiotensin system, and PI3K/Akt signaling was unregulated in CD31+ HemEC micro tumors. The results of drug screening revealed that propranolol had promising therapeutic effects and that metformin had some effects on the micro tumors [113].

## 6 Fabrication of Micro Pattern Array Chips

With the development of microfabrication techniques, physical, chemical, and topographical technologies utilize bioinks (such as carbohydrates, peptides, and proteins with adhesive abilities) to fabricate micro patterns with 2D‐specific shapes on the surface of substrates [32, 213]. These methods can be divided into four main categories: micro contact printing (stamp method), photoresist method, stencil method, and mold method [32, 33]. In addition, an automated micro spotter can generate micropatterns via the transfer of bio inks [125, 136, 160]. Cell fate is determined not only by the bio ink but also by the shape and size of the micro pattern [27]. The shapes and sizes of micro patterns can regulate and limit cell adhesion, geometry, spreading, and migration, which further affect cell proliferation, stiffness, differentiation, and function [33]. The safety, validity, quality, repeatability, and cost of micro patterns guarantee the efficacy of further biomedical applications. Consequently, the fabrication of micro patterns should follow good manufacturing practices (GMPs) [33, 214, 215]. In this section, the use of bio inks as adhesion molecules and culture matrices, the methods used to fabricate micro patterns, and the shapes and the quality of micro patterns are described and discussed (Table 3).

**TABLE 3:** Summary of micro pattern fabrications, cell types, micro pattern shape, and applications in 3D cell culture.

Fabrication methods	Bioinks	Cell types	Micropattern shape	Specific applications	References
Microcontact printing	Collagen I/ IV	HepaRG cells	Circle	Coculture of HepaRG with 3T3	[147]
		HepG2cells	Circle	Studying hypoxic niches	[172, 194]
		U87 cells	Circle	Studying hypoxic niches	[193]
		Insulinoma cells	Circle	Studying cell‐ECM interactions	[188]
	Fibronectin	3T3 cells	Lines	Cell differentiation	[138]
		PIEC cells	Circle	Monoclonal cell culture	[164]
		Insulinoma cells	Circle	Studying cell‐ECM interactions	[188]
		Pancreatic tumor cells	Circle/square/triangle	Efficiently harvesting spheroids	[175]
		HeLa cells	Circle/square	Studying cell‐cell and ECM Interactions; Drug screening; studying tumor spheroid morphology	[30]
		MDA‐MB‐231 cells	Circle/square	Studying mechanical microenvironments and tumor spheroid morphology	[30]
		MCF‐7 cells	Circle/square	Studying mechanical microenvironments and tumor spheroid morphology	[30, 204]
		MCF10A cells	Circle/square	Studying mechanical microenvironments and tumor spheroid morphology	[30, 204]
		A375 cells	Circle/square	Studying tumor spheroid morphology	[30]
		A549 cells	Circle	Studying mechanical microenvironments	[204]
		HepG2 cells	Circle/square	HepG2 spheroid culture	[30]
	Laminin	Insulinoma cells	Circle	Studying cell‐ECM interactions	[188]
	Matrigel	PSCs	Circle/ square	Gut spheroid culture	[112]
	dECM	Primary renal fibroblasts	Circle	Monoclonal cell culture	[116]
		HepG2 cells	Circle/square	Drug screening	[27, 170]
		A549 cells	Circle	Drug screening	[29, 170]
		H1299 cells	Circle	Drug screening	[29]
		CD31+ HemECs	Circle	Drug screening	[113]
		OS‐RC‐2 cells	Circle	OS‐RC‐2 spheroid culture	[170]
	Polydopamine	MSCs	Circle/ square	Efficiently harvesting MSCs spheroids	[117, 134, 143]
		HUVECs	Square	Vascularized MSCs spheroid culture	[134]
		HDFBs	Circle/ square/ lines	Studying mechanism of spheroid formation	[133, 137]
	PolyAA	MCF‐7 cells	Circle	MCF‐7 spheroid culture	[174]
Photoresist method	Collagen I/IV	MSCs	Circle	MSCs spheroid culture and differentiation	[131]
		Primary hepatocytes	Circle	Hepatocyte spheroids culture	[132]
	Fibronectin	PSCs	Circle	Cardiac micro chambers culture	[123]
	Laminin	PSCs	Circle	Studying embryonic development	[126]
	Gelatin	Chinese hamster ovary cells	Circle	Producing recombinant proteins	[165]
		Chondrocytes	Circle	Micro pattern‐noninvasive monitoring system	[166]
	BP nanofibers	HepG2 cells	Circle	HepG2 spheroid culture and detection	[173]
	—	Primary hepatocytes	Circle	Hepatocyte spheroids culture	[144]
		HepG2 cells	Circle	Coculture of HepG2 with 3T3	[186]
		FLC‐4 cells	Circle	Coculture of FLC‐4 with endothelial cells	[187]
Commercial micropatterns	PIPAAm	MSCs	Circle	Spheroids modified by the gene editing	[142]
		Primary hepatocytes	Circle	Spheroids modified by the gene editing	[135, 148, 149]
Automated microspotter	ECM proteins (Collagen/ fibronectin/ laminin)	Primary hepatocytes/ LSECs/ HSCs	Circle	Studying cell‐ECM interactions	[136, 159, 160]
		Liver progenitor cells	Circle	Liver progenitor cell differentiation	[125]
	Poly‐L‐lysine	Myeloma cells	Circle	Studying tumor cell‐CAR‐T cell interactions	[171]

### 6.1 Bioinks

Bioinks can be mainly categorized into bio macromolecules (proteins, carbohydrates, and peptides) and micro molecules (amino acids and chemical substances) [216]. Bio inks not only serve as cell adhesion sites to restrict the region of cell spread but also provide a matrix to support cell growth [27].

Proteins, especially certain ECM proteins (fibronectin, collagen type I, collagen type IV, and laminin), are the most widely used to generate micro patterns [136, 188]. These proteins are primary ECM components that are associated with cell adhesion, growth, migration, and differentiation in development, wound healing, and tumor progression [217]. However, recent studies have indicated that a single type of ECM protein struggles to support and regulate cell viability, differentiation, and function [218]. Compound ECM protein mixtures, such as Matrigel and the dECM, have been shown to provide more favorable matrices to support cells due to the richness of ECM proteins, cytokines, chemokines, and growth factors [57]. In particular, dECM can provide a tissue‐specific matrix and microenvironment [170, 218]. In addition, several micro molecules, such as poly‐L‐lysine, poly (N‐isopropylacrylamide) (PIPAAm), arginine, glycine, aspartic acid, D‐phenylalanine, and polydopamine, which have the ability to adhere to cells and are easy to process, stable, and inexpensive have also been used to generate micro patterns [33, 117, 142, 171]. Regulatory proteins, such as growth factors, can also be used for micro patterning. For example, DNA origami‐based surface patterning with epidermal growth factor (EGF) ligands is a powerful tool to study the mechanism of EGF receptor activation in adherent cells [219].

### 6.2 Fabrication Methods for Micro Patterns

#### 6.2.1 Micro Contact Printing

With the advantages of being cost effective, convenient and easy to use, micro contact printing is one of the most widely used methods to fabricate micro patterns [220]. Generally, silicon wafers with specific patterns are fabricated by laser etching, then used as templates, and PDMS stamps are obtained. The surface of PDMS stamps contains micro‐cylinders. The shape and size of the surface cylinders determine the shape and size of micro patterns [170]. Due to the stronger adhesion between the bio ink and substrates than between the bio ink and PDMS stamps, the bio ink can be transferred from the surface of PDMS stamps to substrates (such as plastics, glass, gold, and hydrogels) to form a patterned matrix [221]. Finally, substrates with micro patterns are treated with bio surfactants (e.g., Pluronic F‐127 or polyethylene glycol) to block regions without micro patterns to prevent nonspecific cellular adhesion [30, 131].

At present, several micro contact printing devices have been developed, some even employing mechanization and automation. Hannachi et al. fabricated a portable micro contact printing device to control the pressure of printing through a piston‐like structure to avoid pressure nonconformity. With its small size and ease of sterilization, this device can control the resolution of printing from 20 to 500 µm [222]. To improve the precision of printing and avoid manual printing, Tanaka et al. employed an automated stamping system that could precisely control a stamping force of 0.1 N to improve the quality of micro patterns through a load cell and an automated actuator [223]. Chakra et al. developed an automated micro contact printing device composed of a stamp holder, a substrate holder, a pneumatic actuator (which controls the movement of the stamp holder and substrate holder through a computer) and a CCD camera (to observe the printing process) to improve the precision of printing and the standardization of manufacturing [224]. Moreover, McNulty et al. reported that a compliant articulated robotic arm could precisely and routinely carry PDMS stamps or substrates to automatically fabricate micro patterns. The precision and accuracy of printing reached 10 µm. In general, the micro contact printing method is suitable for the direct transfer of adhesion molecules to substrates and for blocking the regions of nonspecific cellular adhesion [225].

#### 6.2.2 Photoresist Method

With its high precision and resolution of micro patterns, the photoresist method has become one of the most widely used processing methods for fabricating micro patterns on glass, plastics, cell culture dishes, PDMS, and hydrogels [33, 226]. In the photoresist method, photosensitive materials can be degraded or strengthened by UV light, which is determined by positive or negative photoresists [79, 227]. By using a mask to control the shape and size of the light exposure, photosensitive materials are selectively degraded or strengthened to form specific regions with micro patterns [32]. The shape and size of the mask are related to the shape and size of the micro pattern. Exposed regions can be pre‐coated or subsequently coated with adhesion molecules to block areas of non‐specific cellular adhesion [33]. To date, several lithographic equipment systems (e.g., PRIMO) have been commercialized, accelerating the GMP‐compliant production of micro patterns [228].

#### 6.2.3 Stencil Method

The stencil method is based on the physical limit of directly patterning adhesion molecules or cells. Through‐hole structures control the coating of adhesion molecules to form micro patterns or directly regulate cell seeding on substrates [32]. The morphology is determined by the shape and size of the holes. The stencil method does not require professional facilities, and the equipment can be reused and is easily applied, but it is difficult to control the precision and resolution of micro patterns [33].

#### 6.2.4 Mold Method

Micro contact printing requires complete contact between PDMS stamps and flat substrates, and the photoresist method involves many chemical components that may affect cell culture [33]. The mold method can generate micro patterns on uneven and heterogeneous surfaces and avoids the use of chemical components [101]. The mold method is similar to the stencil method. Elastomeric stamps fabricated from PDMS contain micro channels that are used to selectively coat adhesion molecules. These selectively coated adhesion molecules can be solidified based on their thermo sensitivity or photosensitivity [32, 101]. Becher et al. developed PDMS chips containing 5‐µm channels that were injected with fibronectin. After incubation, the chips were removed, and micro patterns were generated on glass‐bottom dishes [229].

### 6.3 Shapes of Micro patterns

Micro patterns, either for single‐cell or group‐cell patterning, are used to study cell proliferation, apoptosis, differentiation, polarity, migration, development, and morphology, which depend on the shapes of micro patterns [31, 33]. Cell growth, density, adhesion, spreading, and geometry are regulated by the shapes of micro patterns [34].

In 3D cell spheroid culture, the shapes of micro patterns are mainly circular, square, or linear, and the sizes of micropatterns range from microns to millimeters [115, 116]. Regardless of whether non‐tumor or tumor spheroids are cultured, circular micro patterns (diameter 100–200 µm) are most commonly used, followed by square micropatterns. Zhu et al. reported that the efficiency of spheroid generation did not differ between circular and square micro patterns coated on untreated cell culture dishes, while circular micro patterns were more effective in controlling the uniformity of spheroid morphology than were square micro patterns. Moreover, the size of the micro patterns affects spheroid morphology and the microenvironment. As the size of a micro pattern increased, the spheroid morphology was shown to gradually change from a 3D spheroid shape to a hemispherical cap shape [27, 29]. However, for micropatterns coated on thermo sensitive hydrogels, Kim et al. showed that square micro patterns were more beneficial for the formation of spheroids than were circular micro patterns [137]. This difference may be related to different mechanisms of spontaneous spheroid assembly. Moreover, monoclonal cell spheroids are generally cultured on small circular micropatterns (50 µm) [116, 164]. Linear micro patterns, such as HDFBs, are suitable for culturing fibrous microtissues [133].

In general, the shape and size of micro patterns can influence the morphology of spheroids or micro tissues. Therefore, micropatterns of different specifications can be chosen according to the purpose of the study.

### 6.4 Quality of Micro patterns

Generally, the quality of micro patterns is primarily related to the bio inks and manufacturing methods used. Since bio inks provide cell adhesion sites and a specific ECM, the bioactivity of bio inks should be maintained. The storage temperature and the storage time of micro patterns are very important for the bioactivity of bio inks. These conditions are similar to those used for commercial cell culture dishes that are coated with collagen or Matrigel and should be stored at ‐20°C [230]. Consequently, the storage temperature and time are selected according to the bio ink category. With the development of automated equipment, the fabrication of micro patterns has gradually become standardized [27]. However, there are no criteria for micropattern fabrication or reports on the production of GMP‐grade micro patterns. The development of GMP‐grade micropatterns is of great relevance to culturing primary tumors or non‐tumour spheroids for precision medicine and regenerative medicine [33].

## 7 Conclusion and Outlook

For a long time, studies have been devoted to the development of 3D in vitro models to bridge the gap between in vivo and in vitro environments. Compared with 2D models, 3D models have more potential to mimic the in vivo microenvironment and its authenticity [9, 17]. However, both 2D and 3D models have their own value. 2D models are widely used for basic research or drug screening because of their economy and operability [51]. 3D organoid models have unlimited potential for understanding embryonic development, tumor processes, and personalized tumor treatment [5, 45]. With low culture costs, high compatibility with and partial reproducibility of the in vivo tissue microenvironment, cell spheroids have been extensively used in drug discovery, tumor therapy, cell therapy, and basic research [6, 15, 206].

At present, a variety of cell spheroid culture methods (including hanging drops, micro wells, shaking systems, hydrogel‐encapsulated culture, microfluidics, 3D bio printing, and electromagnetic, magnetic, and acoustic forces) and automated culture analysis systems have been developed that govern cell spheroid characteristics, such as the shape, size, 3D density, surface characteristics, the internal microenvironment, and the TME [26, 38, 54]. Each method and system have advantages and disadvantages. It is extremely important to choose a cell spheroid culture method according to the purpose of the research. However, there is still a lack of standardization in the culture of cell spheroids and organoids. Automation is helpful in developing standardized procedures for cell spheroids culture to improve the authenticity, stability, and credibility of experimental results [9, 35, 231]. With the development of materials science, microfluidics and 3D imaging technologies, cell spheroid culture platforms can be combined with these technologies to facilitate the development of multidimensional and automated cell spheroid or organoid culture platforms [9, 74]. By combining multiple cell types and microfluidics, cell spheroids are expected to be able to reconstruct the in vivo microenvironment in vitro [58]. However, although the combination of mixed culture and microfluidics makes cell spheroids more similar to tissue, it also reduces their stability and reproducibility. It is therefore worth investigating how to improve the tissue resemblance and stability of cell models.

Hanging drop, micro well method and Matrigel dome culture are the most common 3D cell culture technologies [15]. Hanging drop culture takes advantage of gravity, cell‐cell adhesion and proliferation to enable cells to aggregate or self‐assemble into spheroids [54]. The micro well method allows cells to be suspended in a limited volume, which increases intercellular interactions, aggregation, and adhesion, leading to the generation of spheroids [6]. Spheroid size and shape can be roughly controlled by seeding a certain number of cells into each droplet or micro well. However, these processes are labor‐intensive and difficult to achieve at a large‐scale with wide diameter variation and irregular morphology of spheroids [27]. Matrigel, which contains various ECM proteins and factors and has a porous structure, provides a biomimetic matrix that induces cell migration, signal transduction among cells, and interactions between cells and the matrix, thereby promoting the self‐aggregation and assembly of cells [230]. Nevertheless, spheroids cultured using the Matrigel dome method exhibit significant heterogeneity in aspects such as morphology, size, and maturity [129]. Compared with these methods, micro patterns can effectively control the size, shape, and arrangement of spheroids or organoids by restricting cell adhesion and growth, which have great potential for use in the standardization of spheroid culture [29, 111]. Moreover, fast and high magnification optical imaging is a prerequisite for high throughput and automated analysis of spheroids and organoids. Compared with classic spheroid and organoid culture techniques, such as suspension culture and Matrigel dome culture, micro pattern technique generated spheroids and organoids have more controllable spatial locations. which enabling AI analyses based on optical micro imaging. This is attributed to the limitation of the working distance in optical imaging, particularly that of high‐magnification objective lenses. In the case of a micro well array, the non‐planar bottom surface of the culture micro wells also poses a limitation to optical imaging at high magnifications. This micro patterned cell culture platform is suitable for large‐scale and standardized culture for regenerative medicine, TME reconstruction, drug screening and automated high‐throughput analysis [29, 117, 133, 143]. Bio inks play an indispensable role in micro patterns. As adhesion molecules, they supply adhesion sites and a specific ECM. Therefore, it is important to develop novel bio inks that can provide multiple specific active molecules and a mechanical microenvironment. Moreover, micro patterns can be combined with microfluidics for achieve dynamic culture, automated dosing, sampling, and control and 3D imaging systems to achieve dynamic observation and automated morphological analysis, which can accelerate the development of one‐chip assays [171, 208, 209]. Micro patterns have also been used in multicellular spheroid culture to develop more tumor spheroid models that mimic the TME. In terms of drug screening, the advantages of micro patterns have been identified [29]. Homogeneous tumor spheroids located at the same level can be combined with 3D imaging systems to reveal drug efficacy and show internal and external changes in spheroid morphology, which are enormously helpful in drug discovery and development [209]. It is urgent to culture clinical samples on micro patterns to generate tumor spheroids/organoids that can be used for drug screening to guide clinical treatment. It is worth noting that in order to promote applications and clinical transformation, the manufacturing techniques of micro patterns should comply with GMP standards.

Based on diverse application scenarios, the shape and size of micro patterns, the types of adhesion matrix, the configurations of microfluidics, the labeling technology, and the imaging systems can all be custom‐designed. Therefore, it is essential to build evaluation criteria for micro patterned cells or tissues that are generated by different platforms. We conclud several important criteria to evaluate the micro patterned cells or tissues. (1) Morphology: morphology is an important and the most accessible parameter for evaluating the development and maturation of spheroids and organoids. High‐throughput optical imaging and image analysis enable us to quickly assess the state of these multicellular structures. For instance, morphological descriptors including circularity, the ratio of the minimum enclosing rectangle area, aspect ratio, peak number, the ratio of the maximum inscribed circle, symmetry ratio, and so on can be used to characterize cell morphology [30]. Additionally, the coefficient of variation (CV) can be utilized to evaluate the consistency of the products [15]; (2) specific biological markers: cells may exhibit heterogeneity within micro patterns, particularly during the process of stem cell differentiation. For instance, ESC colonies can represent different germ layers in a sequential order from the outside in, namely: the trophectoderm, endoderm, mesoderm, and ectoderm [115]. Consequently, immunostaining can be used as a means to assess the cellular phenotypes at specific spatial sites within multicellular structures; and (3) genetics: sampling and analyzing the gene expression of the spheroids and organoids generated by the micro patterns are potential assessment method.

## Conflicts of Interest

The authors declare no conflicts of interests.
